# A bacterial artificial chromosome mouse model of amyotrophic lateral sclerosis manifests ‘space cadet syndrome’ on two FVB backgrounds

**DOI:** 10.1242/dmm.052221

**Published:** 2025-02-13

**Authors:** Sophie E. Badger, Ian Coldicott, Ergita Kyrgiou-Balli, Adrian Higginbottom, Chloé Moutin, Kamallia Mohd Imran, John C. Day, Johnathan Cooper-Knock, Richard J. Mead, James J. P. Alix

**Affiliations:** ^1^ Sheffield Institute for Translational Neuroscience, University of Sheffield, Sheffield S10 1HQ, UK; ^2^ Interface Analysis Centre, School of Physics, University of Bristol, Bristol BS8 1TL, UK; ^3^ Neuroscience Institute, University of Sheffield, Sheffield S10 2TN, UK

**Keywords:** Amyotrophic lateral sclerosis, Frontotemporal dementia, Bacterial artificial chromosome, Mouse model, FVB/N, Space cadet syndrome

## Abstract

*C9orf72*-related amyotrophic lateral sclerosis (ALS)/frontotemporal dementia (FTD) has proven difficult to model in mice. Liu et al. (2016) reported a bacterial artificial chromosome (BAC) transgenic mouse displaying behavioural, motor and pathological abnormalities. This was followed by multiple laboratories independently refuting and confirming phenotypes. A proposed explanation centred on the use of different FVB background lines (from The Jackson Laboratory and Janvier Labs). We studied *C9orf72* BAC mice on both backgrounds and found significantly elevated levels of dipeptide repeat proteins, but no evidence of a transgene-associated phenotype. We observed seizures and a gradual decline in functional performance in transgenic and non-transgenic mice, irrespective of genetic background. The phenotype was in keeping with the so-called ‘space cadet syndrome’. Our findings indicate that the differences previously reported are not due to *C9orf72* status and highlight the importance of using genetic backgrounds that do not confound interpretation of neurodegenerative phenotypes.

## Teaser

**Summary:** A *C9orf72* bacterial artificial chromosome mouse model of amyotrophic lateral sclerosis manifested ‘space cadet syndrome’ on two FVB backgrounds. Genetic backgrounds should thus be considered for their potential to confound neurodegenerative models.

## INTRODUCTION

The most common genetic cause of amyotrophic lateral sclerosis (ALS) and frontotemporal dementia (FTD) is a hexanucleotide (GGGGCC) repeat expansion in the first intron of *C9orf72* (DeJesus-Hernandez et al., 2011; Renton et al., 2011). The underlying mechanism through which disease is triggered remains unclear, with toxic gain of function [for example, through dipeptide repeats (DPRs)] or haploinsufficiency suggested. Although progress towards treating *SOD1* pathogenic variants is being made (Miller et al., 2022), the, so far, less successful gene therapy approach for *C9orf72*-related ALS (Boros et al., 2022) highlights the urgent need for preclinical models for the *C9orf72* variant.

Efforts to understand *C9orf72*-related ALS have been hampered by the lack of a robust mouse model. Several groups have developed *C9orf72* mice using differing approaches (Batra and Lee, 2017). All demonstrated absence of an ALS-type phenotype until Liu et al. (2016), who used a bacterial artificial chromosome (BAC) approach to insert a human-derived transgene. They documented a neurodegenerative phenotype with motor and behavioural abnormalities, matching histopathology, incomplete penetrance and a subgroup of mice with an acute-onset phenotype leading to a shortened lifespan.

The study by Liu et al. (2016) was followed by two contrasting reports. In one, two laboratories independently found no phenotype (Mordes et al., 2020). This was accompanied by a response in which two further groups, plus the original laboratory, reported a neurodegenerative phenotype with increased risk of death (Nguyen et al., 2020a). Suggested reasons for this discrepancy included the housing environment within each unit, methodological distinctions and differences in FVB genetic backgrounds of the mice. Specifically, the mice from The Jackson Laboratory FVB-N/J line appeared to show increased seizures, which could have masked the ALS/FTD phenotype; by contrast, the Janvier Labs FVB-N line showed increased disease penetrance.

In these studies, the two FVB lines were not tested in a single institution, leaving uncertainty around whether the two distinct FVB populations are the cause of the discrepancy. We sought to answer this question through a detailed phenotyping study of both The Jackson Laboratory FVB-N/J (‘JAX’) and Janvier Labs FVB-N (‘Janvier’) *C9orf72* BAC transgenic (C9-BAC) mice, together with non-transgenic (NT) littermates in our unit. Despite an abundance of DPRs in both colonies, we found no clear evidence of a transgene-related ALS/FTD phenotype affecting motor or cognitive domain tests. Similarly, we found no histopathological evidence of ALS/FTD. We did, however, observe seizures, spontaneous death and a general decline in the performance of some motor function tests in both transgenic and NT mice, irrespective of genetic background or transgene status. Our findings highlight the importance of using genetic backgrounds that do not have the potential to confound interpretation through pre-existing neurological phenotypes.

## RESULTS

### JAX and Janvier *C9orf72* BAC transgenic mice show expected repeat expansion sizes and DPRs in tissue

**Fig. 1.**
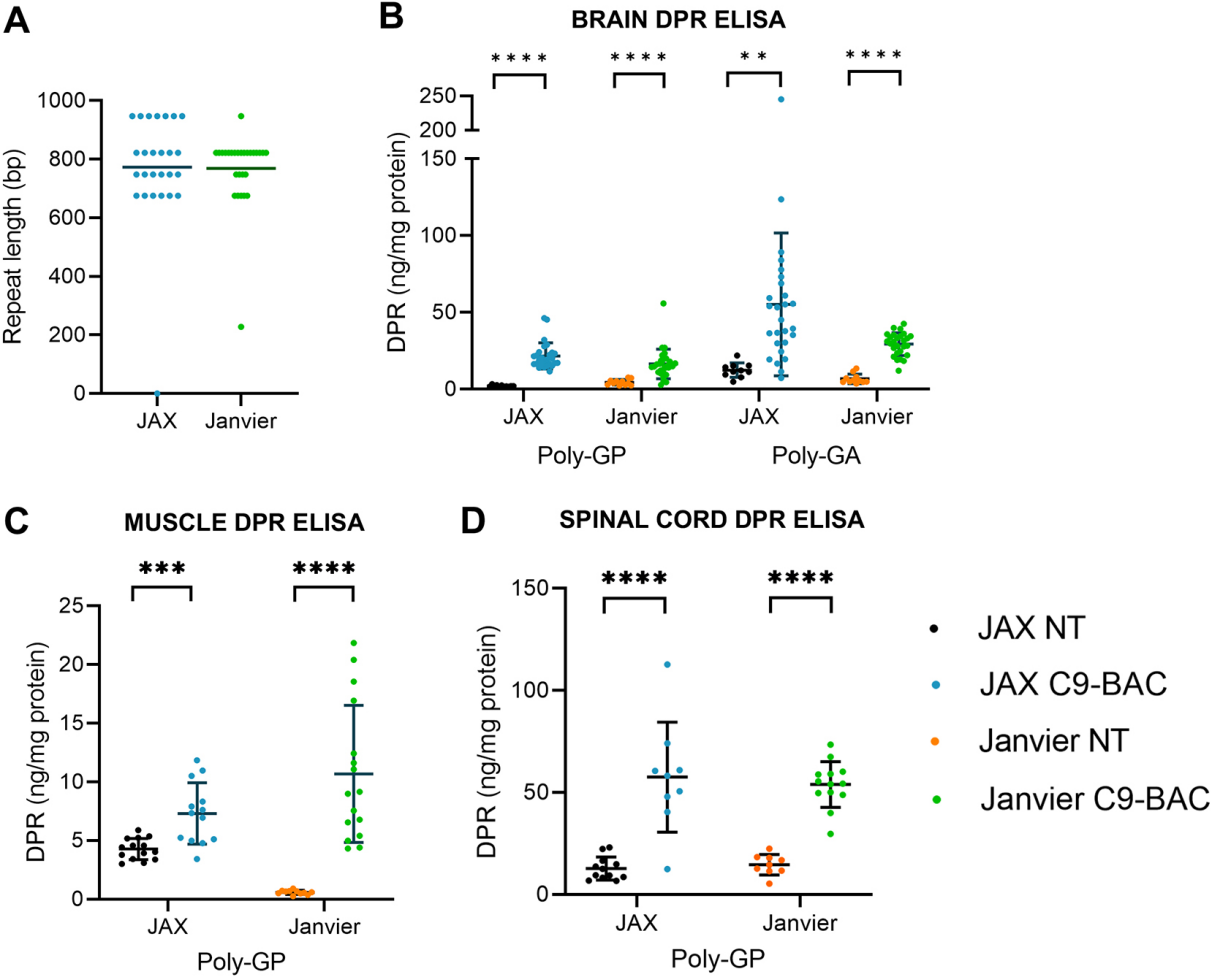
***C9orf72* transgene with GGGGCC repeat expansion size and dipeptide repeat (DPR) protein expression in *C9orf72* bacterial artificial chromosome transgenic (C9-BAC) mice.** (A) Repeat sizes determined by Southern blotting in C9-BAC mice in The Jackson Laboratory FVB-N/J (‘JAX’) and Janvier Labs FVB-N (‘Janvier’) cohorts. One mouse in the Janvier colony had a contraction in repeat length; this mouse was removed from subsequent analyses. (B) Meso Scale Discovery (MSD) immunoassay for poly-GP and poly-GA DPR proteins in the frontal cortex of 52-week-old C9-BAC mice and non-transgenic (NT) littermate controls from both cohorts (JAX C9-BAC, *n*=27; NT, *n* =11; Janvier C9-BAC, *n*=27; NT, *n*=11). (C) MSD immunoassay for poly-GP DPR protein in the gastrocnemius muscle of 52-week-old C9-BAC mice and NT littermate controls from both cohorts (JAX C9-BAC, *n*=13; NT, *n*=14; Janvier C9-BAC, *n*=16; NT, *n*=11). (D) MSD immunoassay for poly-GP DPR protein in the spinal cord of 52-week-old C9-BAC mice and NT littermate controls from both cohorts (JAX C9-BAC, *n*=9; NT, *n*=12; Janvier C9-BAC, *n*=13; NT, *n*=9). Values are mean±s.d.; unpaired two-tailed *t*-test; ****P<0.0001, ***P<0.001, **P<0.01.

The presence of the *C9orf72* BAC transgene and the GGGGCC repeat size was ascertained in the JAX and Janvier colonies through Southern blotting. Both colonies demonstrated expansions between 650 and 950 repeats (Fig. 1). In the JAX colony, two mice dropped the expansion (example in Fig. S1, lane 1); in the Janvier colony, one mouse displayed a contraction in repeat length (Fig. 1). These mice were removed from subsequent data analyses. When the mice reached 1 year of age, we quantified DPR production, observing significantly elevated poly-GP and poly-GA in the frontal cortex of C9-BAC mice from both colonies, but only poly-GP in muscle and spinal cord (Fig. 1B-D).

### No evidence of transgene-specific motor or cognitive phenotypes in JAX C9-BAC or Janvier C9-BAC mice

**Fig. 2.**
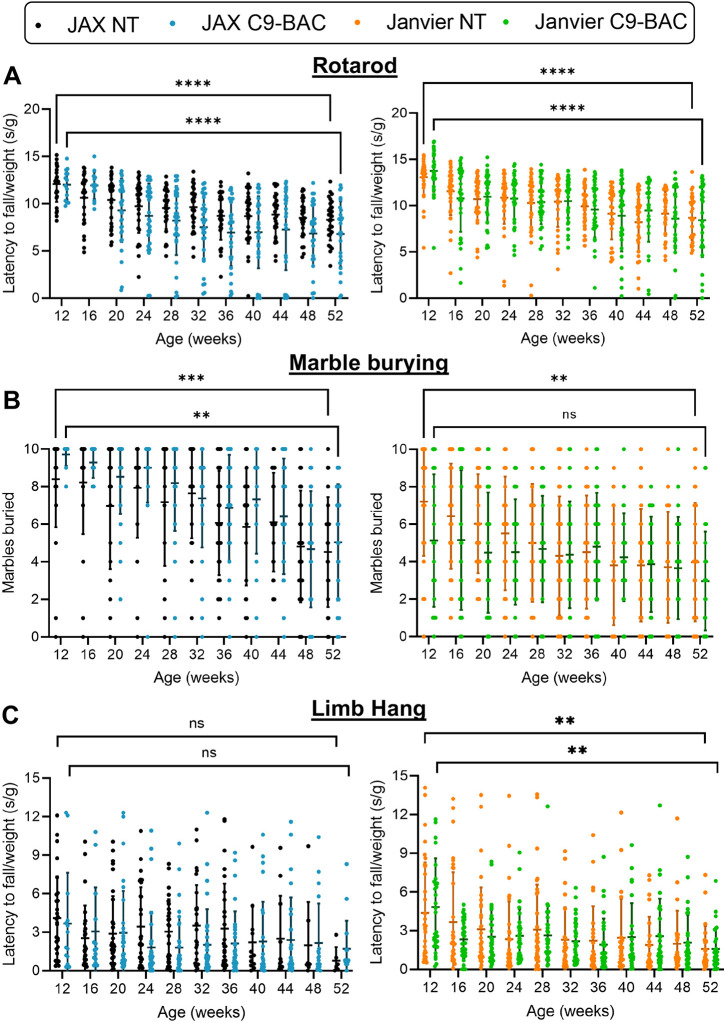
**Absence of motor and motocognitive deficits in C9-BAC mice.** (A) Rotarod performance accounting for weight demonstrated a significant decrease in performance over time in all groups (JAX C9-BAC, *n*=14-32; NT, *n*=27-33; Janvier C9-BAC, *n*=29-32; NT, *n*=28-35). (B) Total number of marbles buried showed significant decrease in all groups except Janvier C9-BAC mice (JAX C9-BAC, *n*=14-32; NT, *n*=27-33; Janvier C9-BAC, *n*=29-32; NT, *n*=28-35). (C) Latency to fall in the limb hang test when accounting for weight revealed no significant decrease in performance over time in the JAX cohort (left), but a significant decrease over time was observed in C9-BAC and NT littermates in the Janvier cohort (right) (JAX C9-BAC, *n*=14-32; NT, *n*=27-33; Janvier C9-BAC, *n*=29-32; NT, *n*=23-35). Values are mean±s.d.; two-way ANOVA with repeated measures and Tukey's post-hoc test; ns, not significant; ****P<0.0001, ***P<0.001, **P<0.01.

As a number of motor and cognitive phenotype abnormalities were reported by Liu et al. (2016) and Nguyen et al. (2020a), we performed a comprehensive phenotyping study over 12 months. We compared C9-BAC to NT mice at 4-weekly intervals, as well as performance over time within each of our four groups (JAX C9-BAC, JAX NT, Janvier C9-BAC, Janvier NT). There were no significant differences between C9-BAC and NT mice in any of our tests at any time points. However, a significant decrease in performance was observed over time in C9-BAC and NT groups in both colonies in the rotarod test (Fig. 2A). A reduction in the number of marbles buried was also seen in C9-BAC and NT JAX mice, and in NT Janvier mice (Fig. 2B). A decline in limb hang performance was observed in C9 BAC and NT Janvier mice (Fig. 2C, right).

**Fig. 3.**
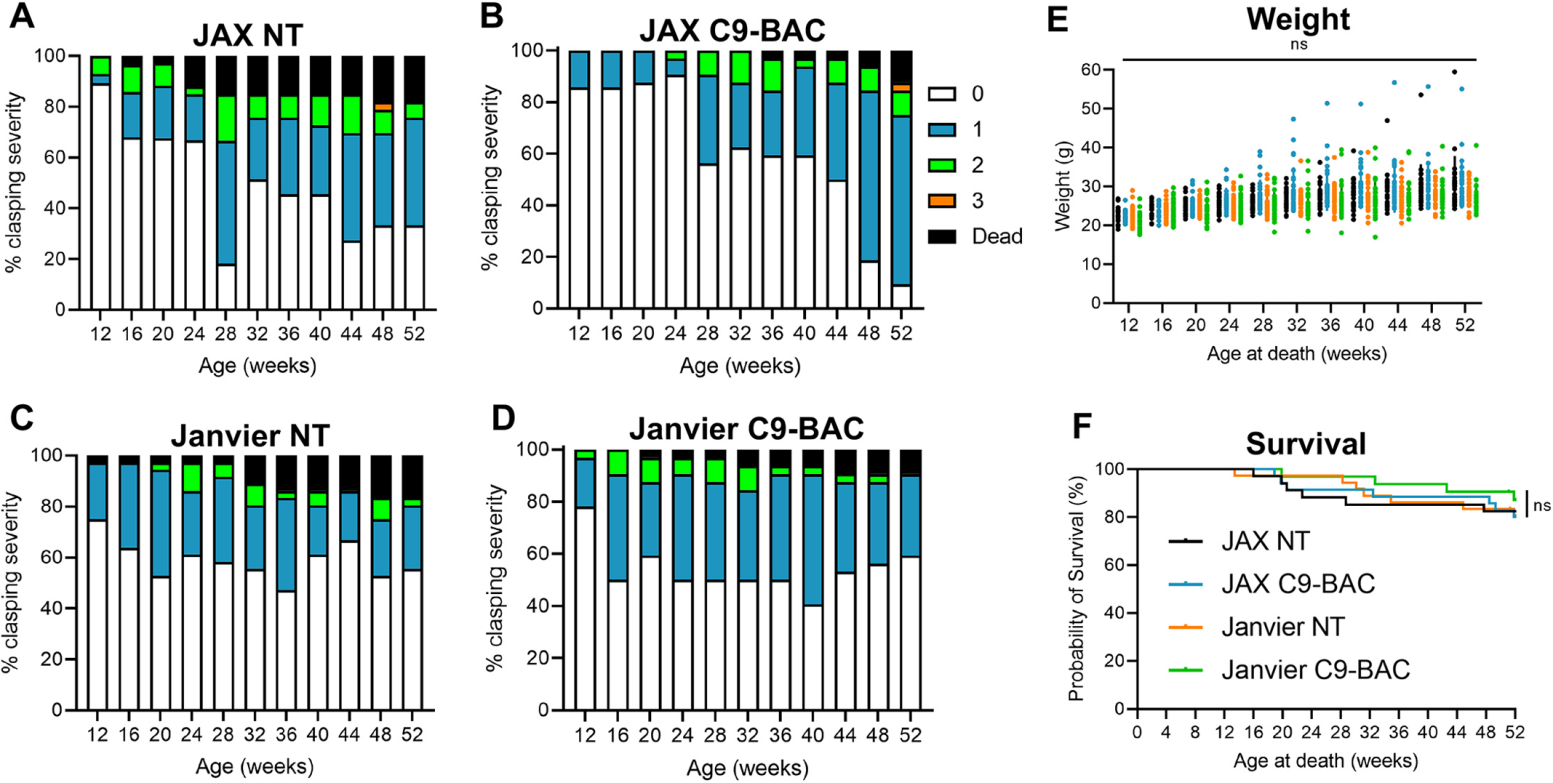
**No clasping, weight loss or reduced survival in C9-BAC mice.** (A-D) Proportions of clasping scores in all mice from JAX and Janvier cohorts [JAX C9-BAC, *n*=35 (B); NT, *n*=34 (A); Janvier C9-BAC, *n*=33 (D); NT, *n*=36 (C)]. (E) Animal weights up to 52 weeks of age (JAX C9-BAC, *n*=14-32; NT, *n*=27-33; Janvier C9-BAC, *n*=29-32; NT, *n*=30-35). Values are mean±s.d.; two-way ANOVA with repeated measures and Tukey's post-hoc test; ns, not significant. (F) Kaplan–Meier plot showing no difference in survival between any of the groups (JAX C9-BAC, *n*=35; NT, *n*=34; Janvier C9-BAC, *n*=33; NT, *n*=36). Values are mean±s.d.; Gehan-Breslow-Wilcoxon test; ns, not significant.

We found no differences in clasping, weight or survival between C9-BAC and NT mice in either colony (Fig. 3). Similarly, no significant longitudinal (or C9-BAC versus NT) differences were seen in open field (Fig. S2), burrowing, nesting, balance beam (Fig. S3) or gait (Fig. S4) tests. Although no differences in social recognition were seen (Fig. S5), a score of >1 indicated that impairment of social memory was evident in C9-BAC and NT mice in both colonies. We investigated the possibility of subsets of mice demonstrating potential ALS/FTD phenotypes but did not find any evidence of this (see Fig. S6 and Table S2). Seizures and spontaneous deaths were observed in C9-BAC and NT mice in both colonies.

### No evidence of brain or spinal cord pathology in JAX C9-BAC or Janvier C9-BAC mice

**Fig. 4.**
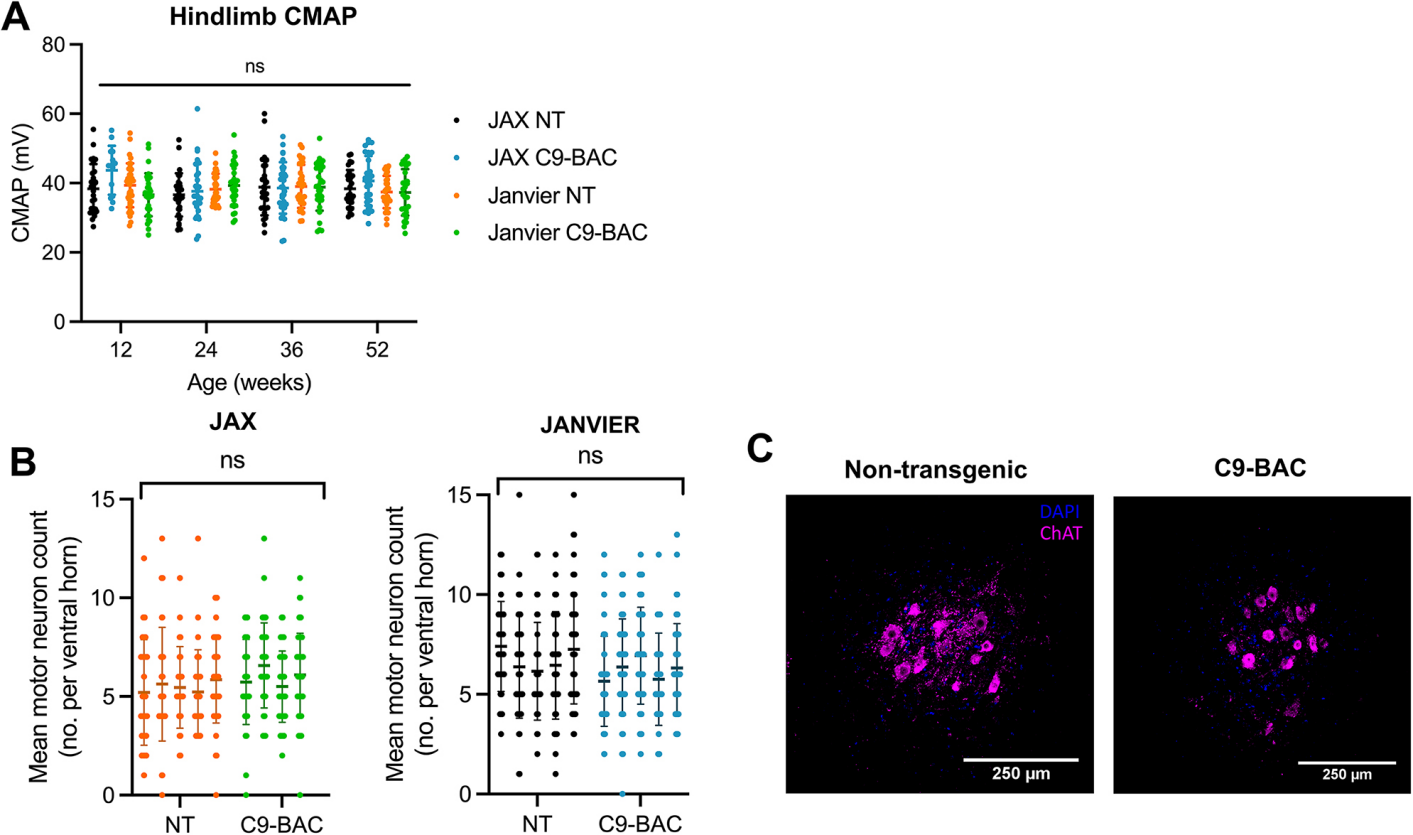
**No motor axon loss or lower motor neuron loss in C9-BAC mice.** (A) Electrophysiology of the hindlimb muscles showed no decrease in compound muscle action potential (CMAP) over time in any group (JAX C9-BAC, *n*=14-32; NT, *n*=28-30; Janvier C9-BAC, *n*=28-32; NT, *n*=28-36). Values are mean±s.d.; repeated measures two-way ANOVA; ns, not significant. (B) Motor neuron counts in ventral horns of the L4-5 region of the spinal cord at 52 weeks of age revealed no significant loss of motor neurons in C9-BAC mice in both cohorts (JAX, *n*=5 per group; Janvier, *n*=5 per group). Values are mean±s.d.; nested *t*-test; ns, not significant. (C) Representative examples of ChAT staining of motor neurons in the ventral horn of the lumbar spinal cord in NT and C9-BAC mice from the JAX cohort.

We assessed lower motor neuron loss *in vivo* using electrophysiology and spectroscopy and *ex vivo* using histology. Hindlimb compound muscle action potentials demonstrated no differences between groups or over time (Fig. 4A). Detailed electrophysiological studies of the neuromuscular junction and lower motor neuron excitability (Fig. S7), as well as molecular spectroscopy of muscle (Fig. S8), revealed no transgene differences. In keeping with these findings, no significant difference in lower motor neuron number was observed between C9-BAC and NT groups in both colonies (Fig. 4B,C; Fig. S9).

**Fig. 5.**
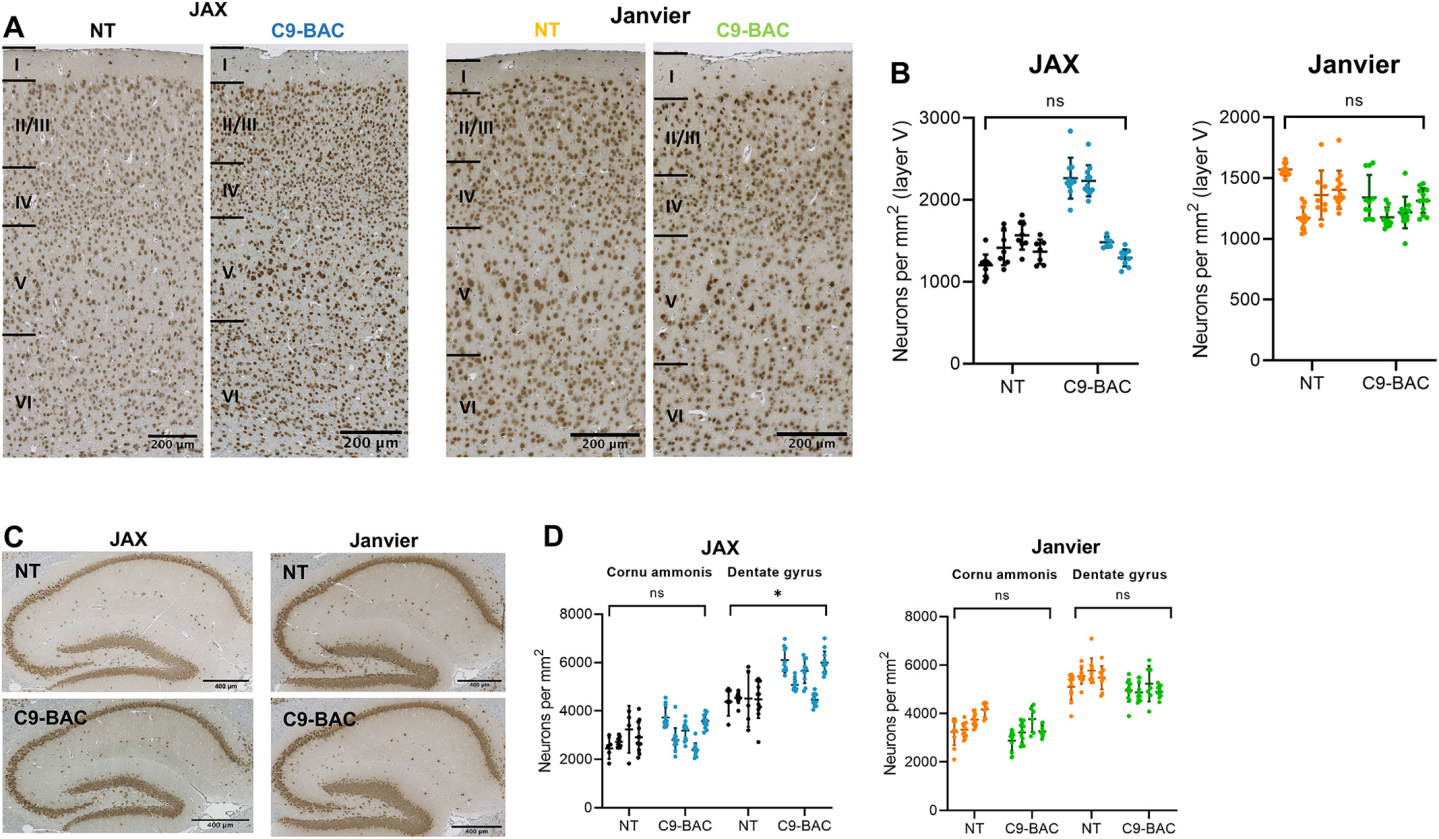
**No motor cortex or hippocampal neuron loss in C9-BAC mice.** (A) Immunohistochemistry with anti-NeuN antibody in C9-BAC and NT mice shows no neurodegeneration in layer V of the motor cortex in either cohort at 52 weeks of age. (B) Quantification of neurons from NeuN-stained sections in layer V of the motor cortex shows no loss of neurons in C9-BAC mice compared to NT littermates in either cohort at 52 weeks of age (*n*=4 mice per group). Values are mean±s.d.; nested one-way ANOVA; ns, not significant. (C) Immunohistochemistry with anti-NeuN antibody in C9-BAC and NT mice shows no transgene-related neurodegeneration in the hippocampus in either cohort at 52 weeks of age. (D) Quantification of neurons from NeuN-stained sections of the hippocampus shows no neurodegeneration in the cornu ammonis or dentate gyrus regions in C9-BAC mice at 52 weeks of age in both cohorts (*n*=4 mice per group). Values are mean±s.d.; nested *t*-test; ns, non-significant; *P<0.05.

At 52 weeks of age, we also assessed cortical pathology. Quantification of NeuN (also known as RBFOX3) staining in the hippocampus and Layer V of the motor cortex revealed no neuronal loss in C9-BAC mice in either cohort (Fig. 5A,B). Hippocampal analysis did not demonstrate neuronal loss in C9-BAC mice (Fig. 5C,D). Additional examinations for the presence of neuroinflammation revealed no evidence of astrogliosis or microgliosis in the dentate gyrus or motor cortex (Figs S10 and S11). Furthermore, there was no evidence of nucleolin displacement (which would indicate nucleolar stress) in the motor cortex of Janvier C9-BAC mice (Fig. S12).

### Neuroinflammation and neuronal loss is associated with the seizure phenotype in the background strain

**Fig. 6.**
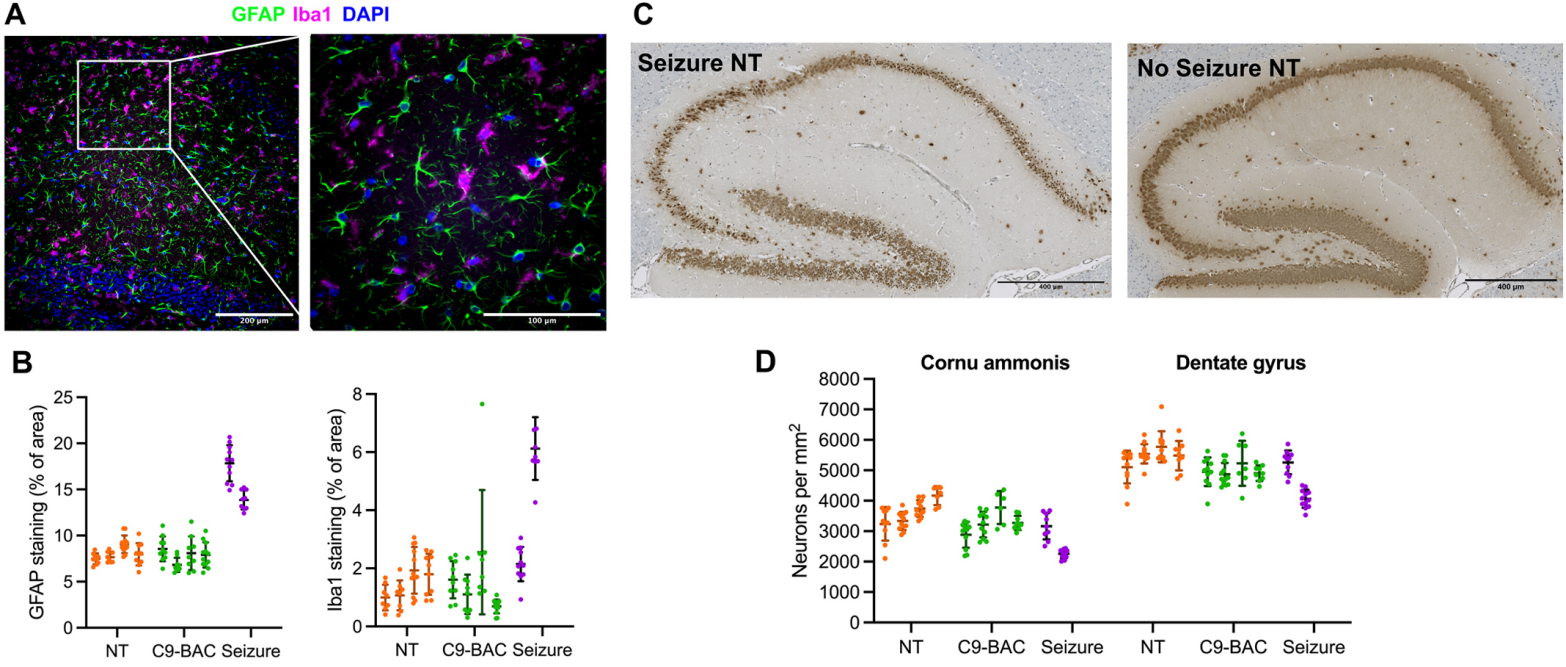
**Pathological changes in the dentate gyrus in NT Janvier mice culled during a seizure.** (A) GFAP and Iba1 staining in the dentate gyrus. Right: 60× magnification of the indicated region on the left. (B) Quantification of GFAP and Iba1 staining shows increased staining area in NT Janvier mice culled mid-seizure (‘Seizure’) in comparison to that in Janvier C9-BAC mice and NT littermates not observed to have seizures (C9-BAC, *n*=4; NT, *n*=4; Seizure, *n*=2). (C) NeuN staining shows neurodegeneration in the cornu ammonis and dentate gyrus regions of the hippocampus in an NT Janvier mouse culled mid-seizure (left) compared to that in an NT mouse not observed to have a seizure (right). (D) Quantification shows neuronal loss in the cornu ammonis and dentate gyrus regions of the hippocampus in NT mice culled mid-seizure compared to that in NT mice not observed to have a seizure (C9-BAC, *n*=4; NT, *n*=4; Seizure, *n*=2).

Several mice in both the JAX and Janvier cohorts were observed to suffer from seizures. In total, we observed seven (10%) mice in the JAX colony to have seizures (five transgenic, two NT), and eight (7%) mice in the Janvier colony (three transgenic, five NT). Histological analyses of two NT mice that were culled mid-seizure showed extensive neuroinflammation in the dentate gyrus, and neuronal loss in both the dentate gyrus and cornu ammonis regions of the hippocampus (Fig. 6). Movies 1 and 2 demonstrate post-ictal behaviour in NT Janvier (Movie 1) and C9-BAC JAX (Movie 2) mice, with minimal responsiveness, signs of distress (orbital narrowing, piloerection, hunched posture) and clasping.

## DISCUSSION

We found no clear evidence of an ALS/FTD phenotype in either the JAX or Janvier FVB colonies, despite relatively stable repeat expansion sizes and the production of DPRs. Our findings are in keeping with those of Mordes et al. (2020) for this mouse model, and with other *C9orf72* repeat expansion BAC transgenic models (Jiang et al., 2016; O'Rourke et al., 2015; Peters et al., 2015).

In both colonies, we saw a gradual decline in performance in several tests (rotarod, marble burying and limb hang), which involve both motor and cognitive components. A decline in related tests was not reported by Mordes et al. (2020) (who failed to detect a transgene-related phenotype), but results similar to ours have been reported previously. For example, a decline in rotarod performance in female wild-type mice after 8 months of age was found by Li et al. (2023). Comparing our social recognition data to results from other strains (Chiang et al., 2018; Farley et al., 2011; Kogan et al., 2000) suggests impairment of social memory, and, in keeping with this, impaired learning/memory function in wild-type FVB mice has also been reported previously (Hsiao et al., 1995). Thus, we have evidence of motor/cognitive defects in both colonies but no evidence that it relates to the inserted *C9orf72* expansion.

In the absence of a transgene effect, what might be causing these deficits? When the FVB background became popular for generating transgenic lines (owing, in part, to large oocyte size), several investigations on the behavioural characteristics of the mice were undertaken. These demonstrated cognitive impairment (Bolivar et al., 2001; Owen et al., 1997), an altered circadian rhythm (Pugh et al., 2004), hyperactivity, anxiety and aggression (Kim et al., 2002; Mineur and Crusio, 2002). Furthermore, female mice, which we used in our work and which manifested the most pronounced phenotype in Liu et al. (2016), appear suspectable to the so-called ‘space cadet syndrome’ (SCS) of impaired social behaviour and sudden death (15-20%) thought to be driven by seizures (Auerbach et al., 2003; Ward et al., 2000). Seizure frequency in female FVB mice has been reported at 17% (Goelz et al., 1998), which is higher than the 7-10% we found across our two colonies. Accurately describing seizure frequency is difficult without sophisticated telemetry systems, and so the true rate of seizures remains unclear. The two Janvier NT mice culled mid-seizure demonstrated astrocyte reactivity, which has also been reported in SCS (Goelz et al., 1998; doi:10.1038/laban0607-16; Mahler et al., 1996). Notably, similar changes were also reported by Liu et al. (2016), highlighting the potential overlap between the reported ALS/FTD and seizure histopathology.

We also examined for neurodegeneration and inflammation. Although there were no significant decreases in neuron counts between C9-BAC mice and NT controls, there was a decreasing trend for neuronal loss in the cornu ammonis and dentate gyrus regions of the hippocampus. This might again relate to SCS, as seizures are associated with hippocampal neurodegeneration in humans (Hattiangady and Shetty, 2008), and recurrent seizures due to SCS or kainic-acid treatment in FVB/N mice have been shown to result in hippocampal neurodegeneration (Goelz et al., 1998; doi:10.1038/laban0607-16; McCord et al., 2008; Silva-Fernandes et al., 2010). The areas of the cornu ammonis that display neurodegeneration in these two NT mice, primarily the CA1 and CA3 regions, are also identical to the areas that showed degeneration in acute C9-BAC mice in the studies by Liu et al. (2016) and Nguyen et al. (2020a). Alongside this, stark increases in astrocyte reactivity and microgliosis were seen in mice culled owing to severe seizures. These findings are comparable to the histopathology reported by Liu et al. (2016) and Nguyen et al. (2020a) in these brain regions, and to the reported SCS in the FVB/N strain (Goelz et al., 1998; doi:10.1038/laban0607-16; Mahler et al., 1996). This makes it challenging to determine whether the pathology seen was indeed caused by the *C9orf72* transgene and highlights how the FVB/N strain might be unsuitable for use in modelling neurodegenerative diseases. Overall, our data are in keeping with the SCS.

The previous literature on the FVB genetic background and our own results highlight the difficulties in working with this strain. It is noteworthy that a prior study examining the effect of amyloid precursor protein (APP) expression in FVB mice documented neophobia and impaired spatial alternation, with diminished glucose utilisation and astrogliosis in the transgenic mice (Hsiao et al., 1995). However, the authors also reported that 20% of their NT mice also developed the same clinical syndrome and postulated that APP overexpression somehow accelerated a naturally occurring age-related disorder in the mice. DPRs have been associated with the development of seizures in other mouse models (LaClair et al., 2020) and *Drosophila* (West et al., 2020). Thus, it could be possible that a similar effect was in action in other laboratories, perhaps driven by factors such as commensal micro-organisms (Burberry et al., 2020).

DPR expression is also worthy of consideration. Only two of the five DPRs were detected in the cortex (poly-GP and poly-GA), and one (poly-GP) was detected in the gastrocnemius muscle and spinal cord; in all tissues only DPRs translated from the sense G_4_C_2_-repeat RNA were identified. Poly-GR, a potentially more ‘toxic’ DPR (Kwon et al., 2014; Mizielinska et al., 2014) was not detected. Poly-GR was not reported by Liu et al. (2016), but, in a subsequent publication, the same group documented poly-GR in the frontal cortex and hindbrain (Nguyen et al., 2020b). The amount detected was very small and at the limit of detection for the Meso Scale Discovery (MSD) enzyme-linked immunosorbent assay (ELISA) we used, so it is possible that we were unable to record it. However, poly-GR was not detected in mice from another laboratory that reported a motor phenotype (Nguyen et al., 2020a).

There were also some methodological differences between our study and that of Nguyen et al. (2020a). For example, in open field testing, Liu et al. (2016) and Mordes et al. (2020) used 30 min trials, whereas we used 10 min trials. Inspection of the data from Liu et al. (2016) shows that mobility was variable. We, like Mordes et al. (2020), found mice within our data to have relatively reduced mobility in this test, with broadly similar performance levels to subsets within the analysis by Liu et al. (2016). However, in both our work and that of Mordes et al. (2020), such performance levels were seen in both C9-BAC and NT mice. Furthermore, Liu et al. (2016) assessed only a small group of C9-BAC and NT mice (NT, *n*=7; asymptomatic C9-BAC, *n*=8; symptomatic C9-BAC, *n*=14) compared to our cohorts (JAX C9-BAC, *n*=14-32; NT, *n*=27-33; Janvier C9-BAC, *n*=29-32; NT, *n*=30-35), which could lead to differences in the representation of poorly performing mice.

However, it should be noted that the above do not entirely account for apparent improvements seen following interventions to target RAN translation such as metformin (Zu et al., 2020) or antibodies (Nguyen et al., 2020b) to target RAN translation. Likewise, the papers of Mordes et al. (2020) and Nguyen et al. (2020a) do not completely resolve the absence/presence of phenotype. What does appear consistent is the presence of anxiety-like behaviour, seizures, gait disturbances, reduced locomotor activity and impaired learning, which have been reported in different laboratories around the world for decades (Bothe et al., 2004; Mineur and Crusio, 2002; Silva-Fernandes et al., 2010). Any change in behaviour/phenotype relating to a transgene must therefore superimpose upon this baseline, which can itself decline over time (Hsiao et al., 1995). Similarly, the reproducibility in preclinical research is a further issue (Justice and Dhillon, 2016; Mandillo et al., 2008), as is the context-dependent biological variation (Voelkl et al., 2020). Each study must therefore be judged with these factors in mind, but, unfortunately, we are unable to provide a definitive explanation as to why they occur.

In summary, we found no evidence of an ALS/FTD phenotype in two cohorts of C9-BAC mice on two different FVB backgrounds. DPR expression provides a molecular fingerprint that could be useful to test DPR-related therapeutic strategies (Castelli et al., 2023), and, in this regard, the model could prove to be a robust platform for studies on this aspect of ALS/FTD pathophysiology. As recently highlighted, it is unrealistic to expect mouse models to manifest all the hallmarks of human disease (Fisher et al., 2023). Perhaps additional insults are required to bring other aspects of the pathophysiology into play and for phenotypes to emerge (Kahriman et al., 2023; Nelson et al., 2024). Further development of *C9orf72* preclinical models, therefore, remains an important area of ALS/FTD research.

## MATERIALS AND METHODS

### Animal model

All mouse experiments were carried out in accordance with the Animals (Scientific Procedures) Act 1986 under a UK Home Office project licence. The ARRIVE Essential reporting guidelines (version 2.0) were followed (Percie du Sert et al., 2020). The *C9orf72* FVB/N model of ALS/FTD generated by Liu et al. (2016) was used in this study. Two separate colonies were generated. The JAX colony was generated using transgenic males containing a human *C9orf72* transgene with 500 hexanucleotide repeats bred to NT FVB/N females from The Jackson Laboratory (stock numbers 029099 and 001800, respectively). The Janvier colony was generated using two transgenic males containing a human *C9orf72* transgene with 500 hexanucleotide repeats donated by Professor Smita Saxena at the University of Bern, Bern, Switzerland, bred to NT FVB/N females from Janvier Labs (stock number SC-FVBN-F). NT littermates were used as controls. Mice were bred in a specified pathogen-free facility and then transferred to a conventional facility for experimental work (12 h light/dark cycle, room temperature of 21°C and 45-55% humidity). Mice were fed 2018 Teklad Global Rodent Diet (Envigo). They were housed in cages of three to five mice but were separated if evidence of fighting was observed. Only female mice were included in these experiments as they exhibited the most severe phenotype in the initial report (Liu et al., 2016). Mice were genotyped for the BAC using C9BAC-forward (5′-TCGAAATGCAGAGAGTGGTG-3′) and C9BAC-reverse (5′-CTTCCTTTCCGGATTATATGTG-3′) primers on DNA isolated from ear clips. A beta-actin control primer was also used (forward, 5′-CTGTCCCTGTATGCCTCTGG-3′; reverse, 5′-AGATGGAGAAAGGACTAGGCTACA-3′). All PCR-positive mice underwent Southern blotting of post-mortem frontal cortex DNA to assess hexanucleotide repeat length. Transgenic mice were excluded from the study if they did not have, or showed a contraction of, the hexanucleotide repeat.

### Southern blotting

DNA was extracted from frontal cortex brain tissue using a DNeasy Blood & Tissue Kit (Qiagen, 69504) following the manufacturer's instructions. A total of 10 µg DNA was digested using Alul and Ddel restriction enzymes (New England Biolabs) at 37°C for 16 h and electrophoresed in a 1% agarose gel in 1× TAE. The agarose gel was denatured in 1.5 M NaCl, 0.5 M NaOH and neutralised in 3 M NaCl, 0.5 M Tris-HCl, pH 7.5. DNA was then transferred to a positively charged nylon membrane (Amersham Hybond N+) by capillary blotting overnight and cross-linked by UV irradiation. A digoxigenin (DIG)-labelled oligonucleotide probe – 5′ [DIG]-(GGGGCC)x5-[DIG] 3′ – was ordered from Thermo Fisher Scientific and denatured at 100°C for 10 min and immediately quenched in ice before use. After 4 h pre-hybridisation in DIG Easy Hyb™ solution (Sigma-Aldrich) at 48°C, the membrane was hybridised with 100 ng/ml probe and fresh DIG Easy Hyb™ solution at 48°C overnight in a rotating hybridisation oven. The membrane was washed with 2× SSC 0.1% SDS at 48-65°C for 10 min, and then washed again with fresh 2× SSC 0.1% SDS for 10 min, 0.5× SSC 0.1% SDS for 15 min, and 0.2× SSC 0.1% SDS for 15 min at 65°C. The membrane was then washed and blocked using the DIG Wash and Block Buffer Set (Sigma-Aldrich) following the manufacturer's instructions, incubated with alkaline phosphatase-conjugated anti-DIG (Merck, 11093274910) at 1:20,000 for 30 min, then washed three times for 10 min in wash buffer. The membrane was then placed in detection buffer followed by application of CSPD ready-to-use chemiluminescent substrate (Roche), sealed in a plastic sheet and exposed for 3 h on chemiluminescent film (Amersham, 28906837).

### DPR quantification

Quantification of DPR protein in the frontal cortex and gastrocnemius muscle was done using MSD ELISA. Tissue was lysed in reporter lysis buffer (Promega) supplemented with 20 µl/ml cOmplete EDTA-free protease inhibitor cocktail (Sigma-Aldrich, 11873580001) and 20 µg/ml PMSF (Sigma-Aldrich, P7626) using a Precellys evolution homogeniser and 1.4 mm zirconium oxide beads (Precellys, 432-0356) at 5500 rpm for 2×30 s, 10 min incubation on ice and another round of homogenisation. Protein lysates were collected after centrifugation at 17,000 ***g*** for 10 min. Protein extract concentrations were determined using a Bradford assay and diluted with lysis buffer to 1 mg/ml or 2 mg/ml for cortex and muscle tissue, respectively, prior to incubation in duplicate with a poly-GP capture antibody (custom synthesis, Eurogentec) coated on 96-well plates. A standard curve was generated for each plate using serial dilutions of the poly-GPx7 peptide CGPGPGPGPGPGPGP in duplicate (0.125 to 40 ng/ml). After washing, a second biotinylated poly-GP detect antibody (generated by Eurogentec from immunisation of a second rabbit) was used with a sulfo-tag streptavidin substrate that led to generation of the electroluminescent signal read in the MSD instrument (following the manufacturer's instructions). The duplicate absorbance readings after blank background subtraction were averaged and converted to concentration unit through the standard curve. Quantifications of poly-GA, poly-GR, poly-PA and poly-PR peptides were performed at the same time in parallel on separate 96-well plates.

### Mouse behavioural tests

All behavioural tests were performed monthly, and electrophysiological tests every 3 months, from 12 weeks to 52 weeks of age. Sample sizes for each test are specified in the figure legends. Sample sizes were determined using previous work with such tests (Mead et al., 2011). Mice were checked daily, and distress was scored weekly; this included assessment for clasping (see Table S1 for details). Seizures were defined as convulsions or behavioural arrest followed by a period of stillness, laboured breathing and lack of response when provoked lasting up to 10 min. Observed seizures were scored according to the scoring system detailed in Van Erum et al. (2019). Severe or repetitive seizures, or failure to recover from seizure, resulted in euthanasia. All tests were performed unaware of genotype.

#### Accelerating rotarod test

Mice were placed on an accelerating rotarod (Ugo Basile, 47500), which gradually rotated from 4 rpm to 40 rpm over a period of 5 min. Each animal underwent two trials with at least 5 min between each trial, and the best performance was used for analysis. Initial training was performed over 3 consecutive days at 12 weeks of age before the first test.

#### Marble-burying test

Individual mice were placed in a cage (42.5 cm×26.6 cm×18.5 cm) filled with 5 cm of sawdust. Ten glass marbles (1 cm diameter) were placed in two columns of five marbles at evenly spaced intervals. Mice were left undisturbed for 30 min under white light before being removed, and the number of marbles buried was counted. A marble was classed as buried when at least two-thirds covered by sawdust.

#### Limb hang

A metal grid was placed 35 cm above a cage (42.5 cm×26.6 cm×18.5 cm) filled with ∼5 cm of sawdust to provide a soft landing. Each mouse was placed on the grid for 3-5 s before the grid was inverted over the cage. The hang period began with all four paws grasping the grid. The hang time was measured from when the grid was inverted to when the mouse fell. Any mouse that jumped off the grid was immediately re-tested. Each mouse underwent two trials, with at least 5 min between each trial, and the average time and the longest time were used for analyses.

### Electrophysiology

Mice were anaesthetised (2% isoflurane), the left hindlimb was shaved, and depilatory cream was used to remove all fur. Recordings were made using a Dantec Keypoint Focus EMG System (Optima, UK). Recordings were made using either needle electrodes (for individual muscle recordings) or ring electrodes (for ‘hindlimb’ recordings). Compound muscle action potentials were obtained via nerve stimulation with a pair of twisted electrodes placed on the skin (square-wave electrical stimulus of 0.1 ms). Supramaximal potentials were obtained for analysis.

### Histology

Mice were deeply anaesthetised with an overdose of pentobarbital and transcardially perfused with phosphate buffered saline (PBS). Brain and spinal cord were dissected and post-fixed in 4% paraformaldehyde for 24 h at 4°C before being stored in PBS at 4°C. Lumbar spinal cord samples were embedded in paraffin wax and then sectioned in the L4-5 region (as per the protocol of Austin et al., 2022) at 10 µm using a microtome and mounted serially on charged slides over a series of five slides, with four sections per slide for a total of 20 slides. For choline acetyltransferase (ChAT) staining, slides were deparaffinised in two changes of xylene and rehydrated through graded ethanol, then antigen retrieved in Reveal Decloaker (Biocare Medical) for a 30 min cycle at 125°C with a pressure of 20 psi. Slides were permeabilised and blocked in 5% bovine serum albumin 0.25% Triton X-100 in PBS for 10 min at room temperature prior to incubation with primary antibody against ChAT (1:100; Merck-Millipore, AB144P) for 72 h at 4°C. Slides were washed three times in PBS and incubated for 1 h at room temperature with donkey anti-goat conjugated to AlexaFluor™ 555 (1:1000, Thermo Fisher Scientific, A28039). Slides were coverslipped using Hardset Vectashield with 4′,6-diamidino-2-phenylindole (DAPI) mounting medium (VectorLabs) and imaged using an IN-Cell Analyzer 2000 (GE Healthcare). Motor neuron counting was performed unaware of genotype using ImageJ software on five animals per group, four slides per mouse, with eight sections per slide. Sections on each slide were separated by 50 µm to prevent double counting. Motor neurons were identified by positive ChAT staining and a clear nucleolus. Any sections with damage or poor staining were excluded. Numbers of motor neurons per ventral horn per section were analysed in GraphPad Prism 9 using a nested *t*-test.

Brain samples were sliced on a mouse brain matrix (Agnthos, Sweden) to isolate either the motor cortex or the hippocampus, embedded in paraffin wax, and sectioned coronally and sagittally, respectively, at 5 µm on a microtome and mounted onto charged slides. Motor cortex and hippocampal sections were mounted continuously, with three sections per slide for a total of 16 slides. NeuN staining was performed by DAB staining using a Vectastain Elite ABC-HRP peroxidase (Rabbit IgG) kit (VectorLabs, PK6101) and a DAB substrate kit (VectorLabs, SK-4100). Sections were deparaffinised and antigen retrieved in access revelation buffer pH 6 (Biocare Medical) for a 30 min cycle at 125°C with a pressure of 20 psi prior to incubation with primary antibody against NeuN (1:500; Cell Signaling Technology, 12943) for 1 h at room temperature. The slides were then washed and incubated with ABC-HRP reagent, washed again and then incubated with DAB substrate, counterstained with Haematoxylin and coverslipped with DPX mounting medium (Cellpath, SEA-1300-00A). Slides were visualised using a digital slide scanner (NanoZoomer XR, Hamamatsu). Neurons were counted in the cornu ammonis and dentate gyrus regions of the hippocampus and layer V of the motor cortex using QuPath's built-in positive cell detection algorithm. Four mice per group, four slides per mouse, with three sections per slide, were counted. Slides were separated by 40 µm to prevent double counting, which was undertaken blind to genotype. Neurons per mm^2^ were analysed in GraphPad Prism 9 using a nested *t*-test.

To assess astrocytosis and microgliosis in the brain, motor cortex and hippocampal sections were immunostained by incubating in primary antibodies against GFAP (1:500; Abcam, ab4674) and Iba1 (also known as AIF1; 1:500; GeneTex, GTX100042) overnight at 4°C and then incubating in appropriate fluorescent secondary antibodies (1:1000; Thermo Fisher Scientific, A11039, A28039) for 90 min. Slides were coverslipped using Hardset Vectashield with 4′,6-diamidino-2-phenylindole (DAPI) mounting medium (VectorLabs) and imaged using an IN-Cell Analyzer 2000 (GE Healthcare). Images were taken at 20× magnification and analysed using ImageJ software. Analysis was performed unaware of genotype. Percentage area staining, integrated density (a measure of staining intensity) and circularity were analysed in GraphPad Prism 9 using a nested *t*-test.

### Statistics

GraphPad Prism 9 was used for all statistical analyses unless otherwise stated. Repeated measure two-way ANOVA with post-hoc Tukey was used for analysis unless otherwise stated. All data are presented as mean±s.d. unless otherwise stated.

## Supplementary Material

10.1242/dmm.052221_sup1 Supplementary information

## References

[DMM052221C1] Auerbach, A. B., Norinsky, R., Ho, W., Losos, K., Guo, Q., Chatterjee, S. and Joyner, A. L. (2003). Strain-dependent differences in the efficiency of transgenic mouse production. *Transgenic Res.* 12, 59-69. 10.1023/a:1022166921766 12650525

[DMM052221C2] Austin, A., Beresford, L., Price, G., Cunningham, T., Kalmar, B. and Yon, M. (2022). Sectioning and counting of motor neurons in the L3 to L6 region of the adult mouse spinal cord. *Curr. Protoc.* 2, e428. 10.1002/cpz1.428 35617451

[DMM052221C3] Batra, R. and Lee, C. W. (2017). Mouse models of C9orf72 hexanucleotide repeat expansion in amyotrophic lateral sclerosis/ frontotemporal dementia. *Front. Cell Neurosci.* 11, 196. 10.3389/fncel.2017.00196 28729824 PMC5498553

[DMM052221C4] Bolivar, V. J., Pooler, O. and Flaherty, L. (2001). Inbred strain variation in contextual and cued fear conditioning behavior. *Mamm. Genome* 12, 651-656. 10.1007/s003350020039 11471061

[DMM052221C5] Boros, B. D., Schoch, K. M., Kreple, C. J. and Miller, T. M. (2022). Antisense Oligonucleotides for the Study and Treatment of ALS. *Neurotherapeutics* 19, 1145-1158. 10.1007/s13311-022-01247-2 35653060 PMC9587169

[DMM052221C6] Bothe, G. W., Bolivar, V. J., Vedder, M. J. and Geistfeld, J. G. (2004). Genetic and behavioral differences among five inbred mouse strains commonly used in the production of transgenic and knockout mice. *Genes Brain Behav.* 3, 149-157. 10.1111/j.1601-183x.2004.00064.x 15140010

[DMM052221C7] Burberry, A., Wells, M. F., Limone, F., Couto, A., Smith, K. S., Keaney, J., Gillet, G., Van Gastel, N., Wang, J. Y., Pietilainen, O. et al. (2020). C9orf72 suppresses systemic and neural inflammation induced by gut bacteria. *Nature* 582, 89-94. 10.1038/s41586-020-2288-7 32483373 PMC7416879

[DMM052221C8] Castelli, L. M., Lin, Y. H., Sanchez-Martinez, A., Gul, A., Mohd Imran, K., Higginbottom, A., Upadhyay, S. K., Markus, N. M., Rua Martins, R., Cooper-Knock, J. et al. (2023). A cell-penetrant peptide blocking C9ORF72-repeat RNA nuclear export reduces the neurotoxic effects of dipeptide repeat proteins. *Sci. Transl. Med.* 15, eabo3823. 10.1126/scitranslmed.abo3823 36857431

[DMM052221C9] Chiang, M. C., Huang, A. J. Y., Wintzer, M. E., Ohshima, T. and McHugh, T. J. (2018). A role for CA3 in social recognition memory. *Behav. Brain Res.* 354, 22-30. 10.1016/j.bbr.2018.01.019 29355673

[DMM052221C10] Dejesus-Hernandez, M., MacKenzie, I. R., Boeve, B. F., Boxer, A. L., Baker, M., Rutherford, N. J., Nicholson, A. M., Finch, N. A., Flynn, H., Adamson, J. et al. (2011). Expanded GGGGCC hexanucleotide repeat in noncoding region of C9ORF72 causes chromosome 9p-linked FTD and ALS. *Neuron* 72, 245-256. 10.1016/j.neuron.2011.09.011 21944778 PMC3202986

[DMM052221C11] Farley, S. J., McKay, B. M., Disterhoft, J. F. and Weiss, C. (2011). Reevaluating hippocampus-dependent learning in FVB/N mice. *Behav. Neurosci.* 125, 871-878. 10.1037/a0026033 22122148 PMC3246014

[DMM052221C12] Fisher, E. M. C., Greensmith, L., Malaspina, A., Fratta, P., Hanna, M. G., Schiavo, G., Isaacs, A. M., Orrell, R. W., Cunningham, T. J. and Arozena, A. A. (2023). Opinion: more mouse models and more translation needed for ALS. *Mol. Neurodegener.* 18, 30. 10.1186/s13024-023-00619-2 37143081 PMC10161557

[DMM052221C13] Goelz, M. F., Mahler, J., Harry, J., Myers, P., Clark, J., Thigpen, J. E. and Forsythe, D. B. (1998). Neuropathologic findings associated with seizures in FVB mice. *Lab. Anim. Sci.* 48, 34-37. 9517887

[DMM052221C14] Hattiangady, B. and Shetty, A. K. (2008). Implications of decreased hippocampal neurogenesis in chronic temporal lobe epilepsy. *Epilepsia* 49, 26-41. 10.1111/j.1528-1167.2008.01635.x PMC3612505 18522598

[DMM052221C16] Hsiao, K. K., Borchelt, D. R., Olson, K., Johannsdottir, R., Kitt, C., Yunis, W., Xu, S., Eckman, C., Younkin, S., Price, D. et al. (1995). Age-related CNS disorder and early death in transgenic FVB/N mice overexpressing Alzheimer amyloid precursor proteins. *Neuron* 15, 1203-1218. 10.1016/0896-6273(95)90107-8 7576662

[DMM052221C17] Jiang, J., Zhu, Q., Gendron, T. F., Saberi, S., McAlonis-Downes, M., Seelman, A., Stauffer, J. E., Jafar-Nejad, P., Drenner, K., Schulte, D. et al. (2016). Gain of toxicity from ALS/FTD-linked repeat expansions in C9ORF72 is alleviated by antisense oligonucleotides targeting GGGGCC-containing RNAs. *Neuron* 90, 535-550. 10.1016/j.neuron.2016.04.006 27112497 PMC4860075

[DMM052221C18] Justice, M. J. and Dhillon, P. (2016). Using the mouse to model human disease: increasing validity and reproducibility. *Dis. Model. Mech.* 9, 101-103. 10.1242/dmm.024547 26839397 PMC4770152

[DMM052221C19] Kahriman, A., Bouley, J., Tuncali, I., Dogan, E. O., Pereira, M., Luu, T., Bosco, D. A., Jaber, S., Peters, O. M., Brown, R. H. et al. (2023). Repeated mild traumatic brain injury triggers pathology in asymptomatic C9ORF72 transgenic mice. *Brain* 146, 5139-5152. 10.1093/brain/awad264 37527465 PMC11046056

[DMM052221C20] Kim, S., Lee, S., Ryu, S., Suk, J. and Park, C. (2002). Comparative analysis of the anxiety-related behaviors in four inbred mice. *Behav. Processes* 60, 181-190. 10.1016/s0376-6357(02)00085-2 12426069

[DMM052221C21] Kogan, J. H., Frankland, P. W. and Silva, A. J. (2000). Long-term memory underlying hippocampus-dependent social recognition in mice. *Hippocampus* 10, 47-56. 10706216 10.1002/(SICI)1098-1063(2000)10:1<47::AID-HIPO5>3.0.CO;2-6

[DMM052221C22] Kwon, I., Xiang, S., Kato, M., Wu, L., Theodoropoulos, P., Wang, T., Kim, J., Yun, J., Xie, Y. and McKnight, S. L. (2014). Poly-dipeptides encoded by the C9orf72 repeats bind nucleoli, impede RNA biogenesis, and kill cells. *Science* 345, 1139-1145. 10.1126/science.1254917 25081482 PMC4459787

[DMM052221C23] Laclair, K. D., Zhou, Q., Michaelsen, M., Wefers, B., Brill, M. S., Janjic, A., Rathkolb, B., Farny, D., Cygan, M., De Angelis, M. H. et al. (2020). Congenic expression of poly-GA but not poly-PR in mice triggers selective neuron loss and interferon responses found in C9orf72 ALS. *Acta Neuropathol.* 140, 121-142. 10.1007/s00401-020-02176-0 32562018 PMC7360660

[DMM052221C24] Li, S. H., Colson, T.-L. L., Chen, J., Abd-Elrahman, K. S. and Ferguson, S. S. G. (2023). Comparison of Huntington's disease phenotype progression in male and female heterozygous FDNQ175 mice. *Mol. Brain* 16, 67. 10.1186/s13041-023-01054-6 37726802 PMC10508000

[DMM052221C25] Liu, Y., Pattamatta, A., Zu, T., Reid, T., Bardhi, O., Borchelt, D. R., Yachnis, A. T. and Ranum, L. P. (2016). C9orf72 BAC mouse model with motor deficits and neurodegenerative features of ALS/FTD. *Neuron* 90, 521-534. 10.1016/j.neuron.2016.04.005 27112499

[DMM052221C26] Mahler, J. F., Stokes, W., Mann, P. C., Takaoka, M. and Maronpot, R. R. (1996). Spontaneous lesions in aging FVB/N mice. *Toxicol. Pathol.* 24, 710-716. 10.1177/019262339602400606 8994298

[DMM052221C27] Mandillo, S., Tucci, V., Hölter, S. M., Meziane, H., Banchaabouchi, M. A., Kallnik, M., Lad, H. V., Nolan, P. M., Ouagazzal, A. M., Coghill, E. L. et al. (2008). Reliability, robustness, and reproducibility in mouse behavioral phenotyping: a cross-laboratory study. *Physiol. Genomics* 34, 243-255. 10.1152/physiolgenomics.90207.2008 18505770 PMC2519962

[DMM052221C28] McCord, M. C., Lorenzana, A., Bloom, C. S., Chancer, Z. O. and Schauwecker, P. E. (2008). Effect of age on kainate-induced seizure severity and cell death. *Neuroscience* 154, 1143-1153. 10.1016/j.neuroscience.2008.03.082 18479826 PMC2481509

[DMM052221C29] Mead, R. J., Bennett, E. J., Kennerley, A. J., Sharp, P., Sunyach, C., Kasher, P., Berwick, J., Pettmann, B., Battaglia, G., Azzouz, M. et al. (2011). Optimised and rapid pre-clinical screening in the SOD1^G93A^ transgenic mouse model of amyotrophic lateral sclerosis (ALS). *PLoS ONE* 6, e23244. 10.1371/journal.pone.0023244 21876739 PMC3158065

[DMM052221C30] Miller, T. M., Cudkowicz, M. E., Genge, A., Shaw, P. J., Sobue, G., Bucelli, R. C., Chiò, A., Van Damme, P., Ludolph, A. C., Glass, J. D. et al. (2022). Trial of antisense oligonucleotide tofersen for SOD1 ALS. *N. Engl. J. Med.* 387, 1099-1110. 10.1056/NEJMoa2204705 36129998

[DMM052221C31] Mineur, Y. S. and Crusio, W. E. (2002). Behavioral and neuroanatomical characterization of FVB/N inbred mice. *Brain Res. Bull.* 57, 41-47. 10.1016/s0361-9230(01)00635-9 11827736

[DMM052221C32] Mizielinska, S., Gronke, S., Niccoli, T., Ridler, C. E., Clayton, E. L., Devoy, A., Moens, T., Norona, F. E., Woollacott, I. O. C., Pietrzyk, J. et al. (2014). C9orf72 repeat expansions cause neurodegeneration in Drosophila through arginine-rich proteins. *Science* 345, 1192-1194. 10.1126/science.1256800 25103406 PMC4944841

[DMM052221C33] Mordes, D. A., Morrison, B. M., Ament, X. H., Cantrell, C., Mok, J., Eggan, P., Xue, C., Wang, J. Y., Eggan, K. and Rothstein, J. D. (2020). Absence of survival and motor deficits in 500 repeat C9ORF72 BAC mice. *Neuron* 108, 775-783.e4. 10.1016/j.neuron.2020.08.009 33022228

[DMM052221C34] Nelson, A. T., Cicardi, M. E., Markandaiah, S. S., Han, J. Y., Philp, N. J., Welebob, E., Haeusler, A. R., Pasinelli, P., Manfredi, G., Kawamata, H. et al. (2024). Glucose hypometabolism prompts RAN translation and exacerbates C9orf72-related ALS/FTD phenotypes. *EMBO Rep.* 25, 2479-2510. 10.1038/s44319-024-00140-7 38684907 PMC11094177

[DMM052221C35] Nguyen, L., Laboissonniere, L. A., Guo, S., Pilotto, F., Scheidegger, O., Oestmann, A., Hammond, J. W., Li, H., Hyysalo, A., Peltola, R. et al. (2020a). Survival and motor phenotypes in FVB C9-500 ALS/FTD BAC transgenic mice reproduced by multiple labs. *Neuron* 108, 784-796.e3. 10.1016/j.neuron.2020.09.009 33022226 PMC8281452

[DMM052221C36] Nguyen, L., Montrasio, F., Pattamatta, A., Tusi, S. K., Bardhi, O., Meyer, K. D., Hayes, L., Nakamura, K., Banez-Coronel, M., Coyne, A. et al. (2020b). Antibody therapy targeting RAN proteins rescues C9 ALS/FTD phenotypes in C9orf72 mouse model. *Neuron* 105, 645-662.e11. 10.1016/j.neuron.2019.11.007 31831332 PMC7391607

[DMM052221C37] O'Rourke, J. G., Bogdanik, L., Muhammad, A., Gendron, T. F., Kim, K. J., Austin, A., Cady, J., Liu, E. Y., Zarrow, J., Grant, S. et al. (2015). C9orf72 BAC transgenic mice display typical pathologic features of ALS/FTD. *Neuron* 88, 892-901. 10.1016/j.neuron.2015.10.027 26637796 PMC4672384

[DMM052221C38] Owen, E. H., Logue, S. F., Rasmussen, D. L. and Wehner, J. M. (1997). Assessment of learning by the Morris water task and fear conditioning in inbred mouse strains and F1 hybrids: implications of genetic background for single gene mutations and quantitative trait loci analyses. *Neuroscience* 80, 1087-1099. 10.1016/s0306-4522(97)00165-6 9284062

[DMM052221C39] Percie Du Sert, N., Ahluwalia, A., Alam, S., Avey, M. T., Baker, M., Browne, W. J., Clark, A., Cuthill, I. C., Dirnagl, U., Emerson, M. et al. (2020). Reporting animal research: explanation and elaboration for the ARRIVE guidelines 2.0. *PLoS Biol.* 18, e3000411. 10.1371/journal.pbio.3000411 32663221 PMC7360025

[DMM052221C40] Peters, O. M., Cabrera, G. T., Tran, H., Gendron, T. F., McKeon, J. E., Metterville, J., Weiss, A., Wightman, N., Salameh, J., Kim, J. et al. (2015). Human C9ORF72 hexanucleotide expansion reproduces RNA foci and dipeptide repeat proteins but not neurodegeneration in BAC transgenic mice. *Neuron* 88, 902-909. 10.1016/j.neuron.2015.11.018 26637797 PMC4828340

[DMM052221C41] Pugh, P. L., Ahmed, S. F., Smith, M. I., Upton, N. and Hunter, A. J. (2004). A behavioural characterisation of the FVB/N mouse strain. *Behav. Brain Res.* 155, 283-289. 10.1016/j.bbr.2004.04.021 15364488

[DMM052221C42] Renton, A. E., Majounie, E., Waite, A., Simon-Sanchez, J., Rollinson, S., Gibbs, J. R., Schymick, J. C., Laaksovirta, H., Van Swieten, J. C., Myllykangas, L. et al. (2011). A hexanucleotide repeat expansion in C9ORF72 is the cause of chromosome 9p21-linked ALS-FTD. *Neuron* 72, 257-268. 10.1016/j.neuron.2011.09.010 21944779 PMC3200438

[DMM052221C43] Silva-Fernandes, A., Oliveira, P., Sousa, N. and Maciel, P. (2010). Motor and behavioural abnormalities associated with persistent spontaneous epilepsy in the fvb/n mouse strain. *Scand. J. Lab. Anim. Sci.* 37, 213-222.

[DMM052221C44] Van Erum, J., Van Dam, D. and De Deyn, P. P. (2019). PTZ-induced seizures in mice require a revised Racine scale. *Epilepsy Behav.* 95, 51-55. 10.1016/j.yebeh.2019.02.029 31026782

[DMM052221C45] Voelkl, B., Altman, N. S., Forsman, A., Forstmeier, W., Gurevitch, J., Jaric, I., Karp, N. A., Kas, M. J., Schielzeth, H., Van De Casteele, T. et al. (2020). Reproducibility of animal research in light of biological variation. *Nat. Rev. Neurosci.* 21, 384-393. 10.1038/s41583-020-0313-3 32488205

[DMM052221C46] Ward, J. M., Anver, M. R., Mahler, J. F. and Devor-Hennemann, D. E. (2000). Pathology of mice commonly used in genetic engineering (C57BL/6; 129; B6, 129; and FVB/N). In *Pathology of Genetically Engineered Mice* (ed. J. M. Ward, J. F. Mahler, R. R. Maronpot and J. P. Sundberg), pp. 161-179. Ames, IA: Iowa State University Press.

[DMM052221C47] West, R. J. H., Sharpe, J. L., Voelzmann, A., Munro, A. L., Hahn, I., Baines, R. A. and Pickering-Brown, S. (2020). Co-expression of C9orf72 related dipeptide-repeats over 1000 repeat units reveals age- and combination-specific phenotypic profiles in Drosophila. *Acta Neuropathol. Commun* 8, 158. 10.1186/s40478-020-01028-y 32894207 PMC7487709

[DMM052221C48] Zu, T., Guo, S., Bardhi, O., Ryskamp, D. A., Li, J., Khoramian Tusi, S., Engelbrecht, A., Klippel, K., Chakrabarty, P., Nguyen, L. et al. (2020). Metformin inhibits RAN translation through PKR pathway and mitigates disease in C9orf72 ALS/FTD mice. *Proc. Natl. Acad. Sci. USA* 117, 18591-18599. 10.1073/pnas.2005748117 32690681 PMC7414156

