# Supplementary material for: A bacterial artificial chromosome mouse model of amyotrophic lateral sclerosis manifests ‘space cadet syndrome’ on two FVB backgrounds
Source: Dis Model Mech. 2025 Feb 13;18(2):DMM052221. doi: 10.1242/dmm.052221 (PMC11849976; doi:10.1242/dmm.052221)
Supplement: Supplementary information [file dmm-18-052221-s1.pdf]

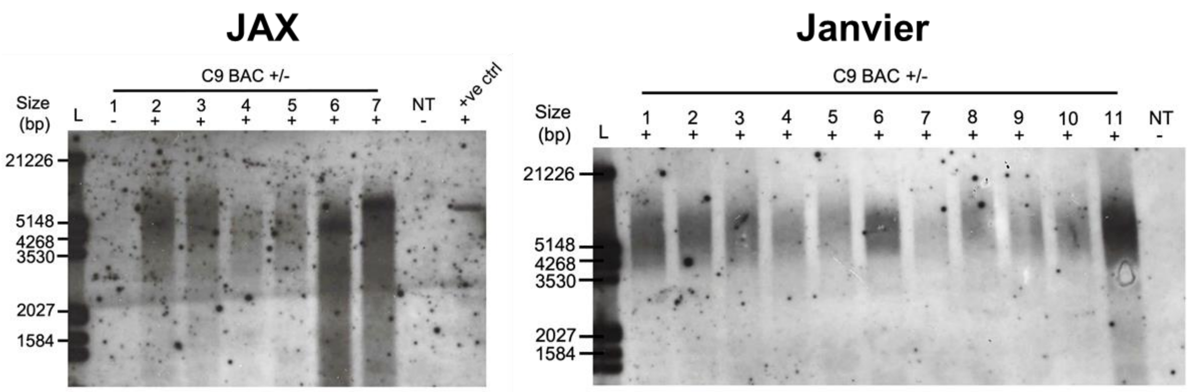

Fig. S1. Southern blot of C9-BAC mice in JAX and Janvier cohorts

Representative examples of southern blots from the JAX and Janvier cohorts (L, DNA ladder; +, repeat present; -, repeat absent; NT, non-transgenic control; +ve ctrl, pcDNA3.1/ G4C2x45 - 3xV5 plasmid with a size of 5,927bp when linearised. Two mice in the Jackson colony dropped the expansion. In the Janvier colony, one mouse displayed a retraction in repeat length. These mice were removed from subsequent data analyses.

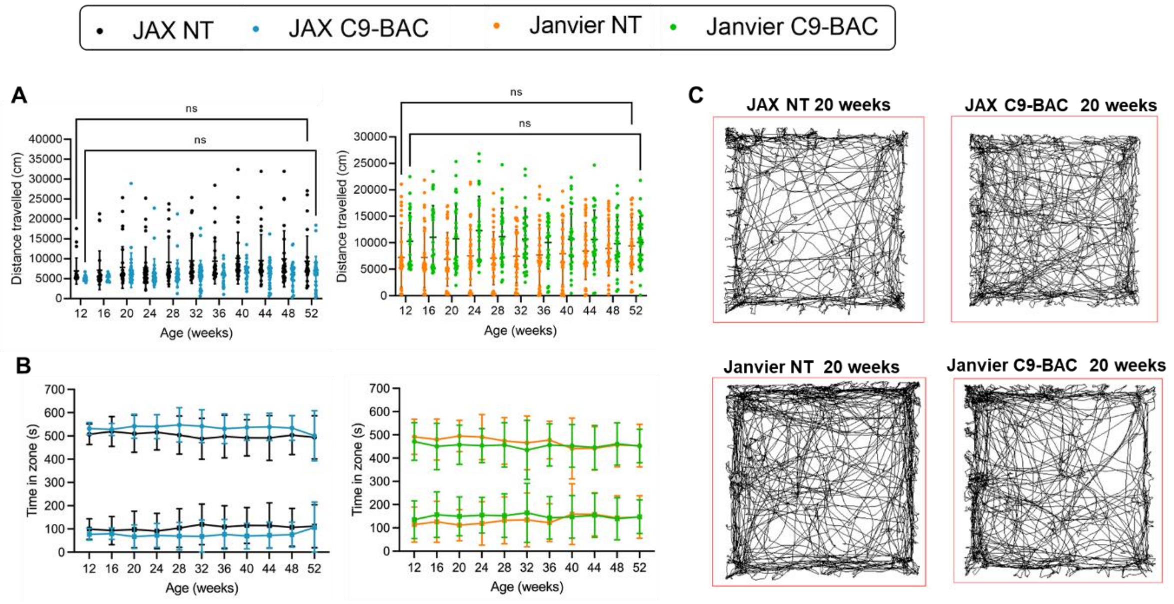

Fig. S2. No Motocognitive deficits observed in C9-BAC mice

A) Total distance travelled in open field shows no change over time in all groups (JAX C9-BAC n = 14-32; NT n = 27-33; Janvier C9-BAC n = 29-32; NT = 30-35).

B) Total time spent in the centre and periphery zones of the open field chamber shows no significantly decreased time spent in the centre in C9-BAC mice compared to NT littermates in both cohorts (JAX C9-BAC n = 14-32; NT n = 27-33; Janvier C9-BAC n = 29-32; NT = 30-35). Square icon = centre, circle icon = periphery.

C) Example open field traces.

Values are mean ± SD.

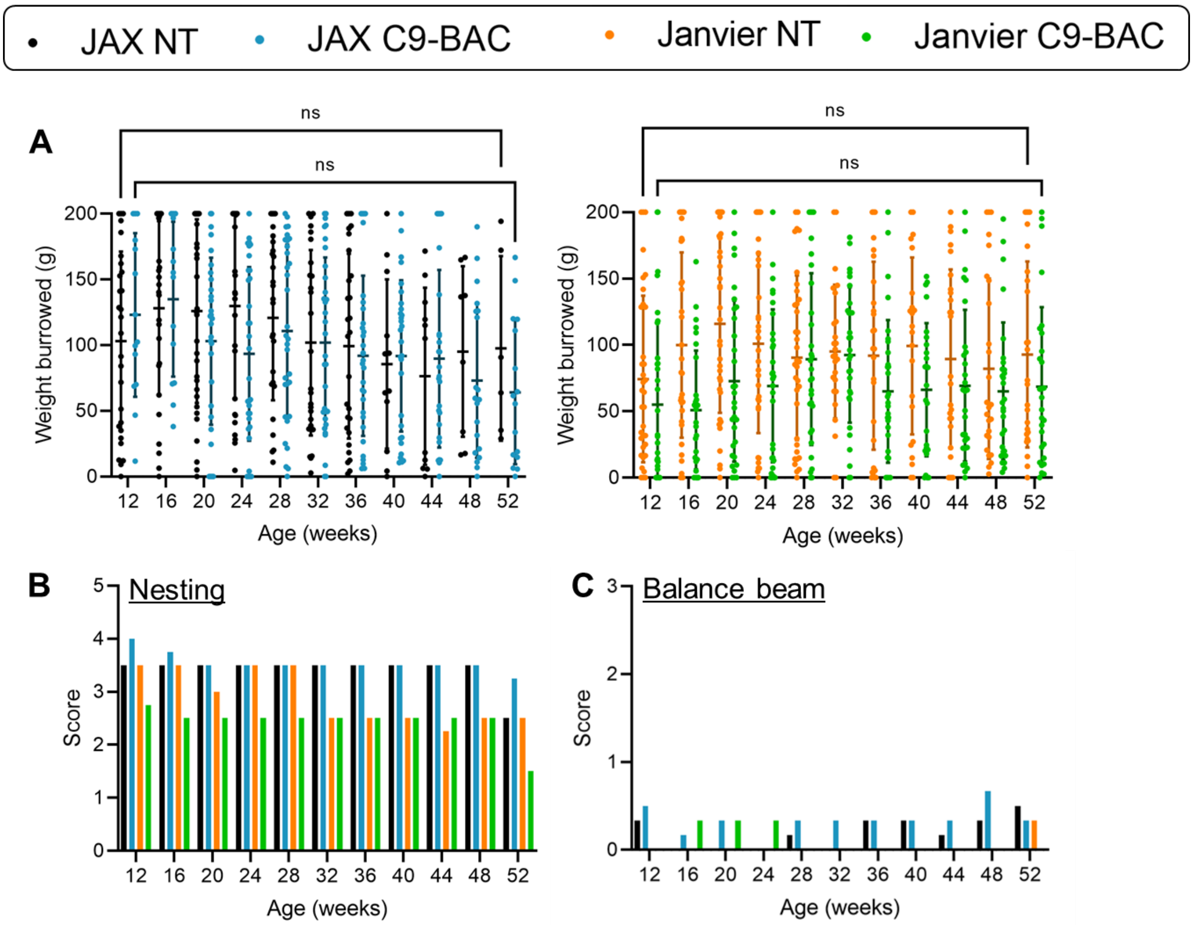

Fig. S3. Absence of motor, cognitive, and motocognitive deficits in C9-BAC mice

A) Weight burrowed shows no significant change over time (JAX C9-BAC n = 14-32; NT n = 27-33; Janvier C9-BAC n = 29-32; NT = 22-35; mean ± SD; two-way ANOVA with repeated measures; ns, non-significant).

B) Median nest scores of both cohorts (JAX C9-BAC n = 14-32; NT n = 27-33; Janvier C9-BAC n = 29-32; NT = 26-35).

C) Median balance beam scores of both cohorts (JAX C9-BAC n = 14-32; NT n = 27-33; Janvier C9-BAC n = 29-32; NT = 26-35).

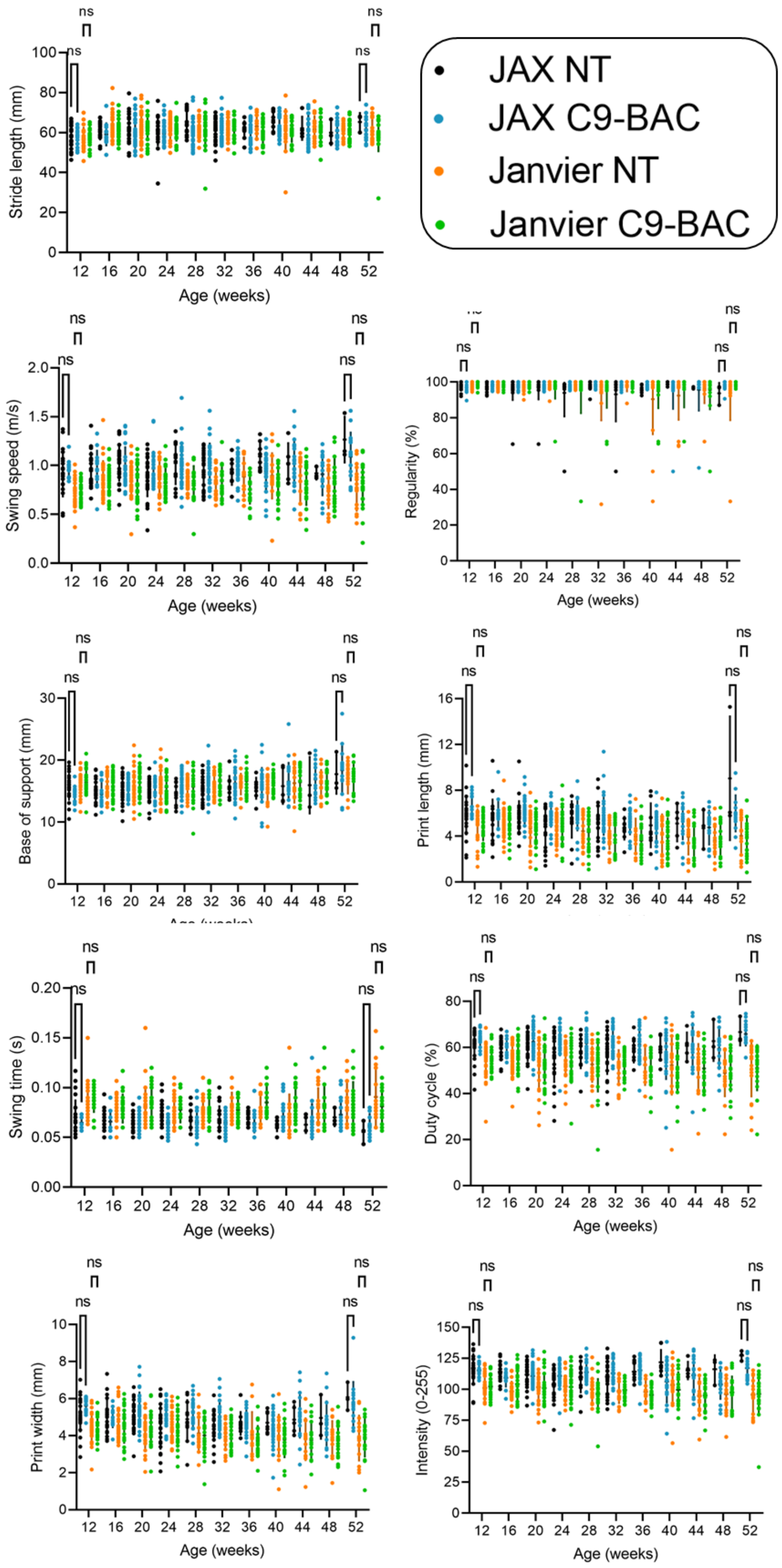
JAX NT
JAX C9-BAC
Janvier NT
Janvier C9-BAC
ns
Stride length (mm)
Swing speed (m/s)
Regularity (%)
Base of support (mm)
Print length (mm)
Swing time (s)
Duty cycle (%)
Print width (mm)
Intensity (0-255)
Age (weeks)
12 16 20 24 28 32 36 40 44 48 52

Fig. S4. No gait abnormalities in C9-BAC mice.

No significant differences observed between C9-BAC and non-transgenic littermates in any of the catwalk gait analysis parameters analysed in either cohort (JAX C9-BAC n = 14-30; NT n = 3-28; Janvier C9-BAC n = 21-28; NT n = 12-33).

Values are mean ± SD; two-way ANOVA with repeated measures; ns, not significant.

Disease Models & Mechanisms • Supplementary information

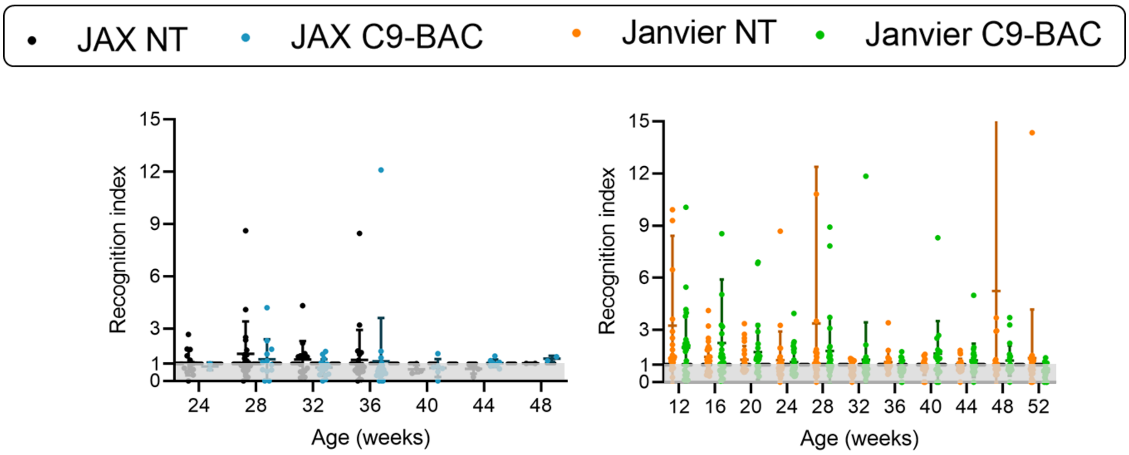

Fig. S5. No transgene related impairment of social recognition.

Recognition index between recall and initial sessions demonstrates inability to recall a familiar conspecific in all groups.

The shaded area represents a score of ≤1, which indicates either the same or less time spent investigating an unfamiliar mouse relative to a familiar mouse (JAX C9-BAC n = 2-31; NT n = 2-28; Janvier C9-BAC n = 19-30; NT = 14-28).

Disease Models & Mechanisms • Supplementary information

Fig. S6. Subgroup analysis

Liu et al. (2016) reported that a subset of mice developed an acute, progressive phenotype. We investigated the presence of subsets of mice by defining changes in performance for each test that could be compatible with a neurodegenerative phenotype; namely, a reduction in performance level that either plateaued or progressed. Example data patterns are shown.

We considered scores 2, 6 7 and 9 (shaded) as profiles potentially compatible with a neurodegenerative ALS-FTD phenotype, representing consistent dysfunction or a deterioration in function without improvement.

Code	Code description
1	Stable: In the same quartile throughout
2	Stable poor: Consistently in the bottom quartile
3	Stable good: Consistently in the top quartile
4	Increase (change quartile) then stable
5	Increase (change quartile) then increase (change quartile)
6	Increase (change quartile) then decrease (change quartile)
7	Decrease (change quartile) then stable
8	Decrease (change quartile) then increase (change quartile)
9	Decrease (change quartile) then decrease (change quartile)

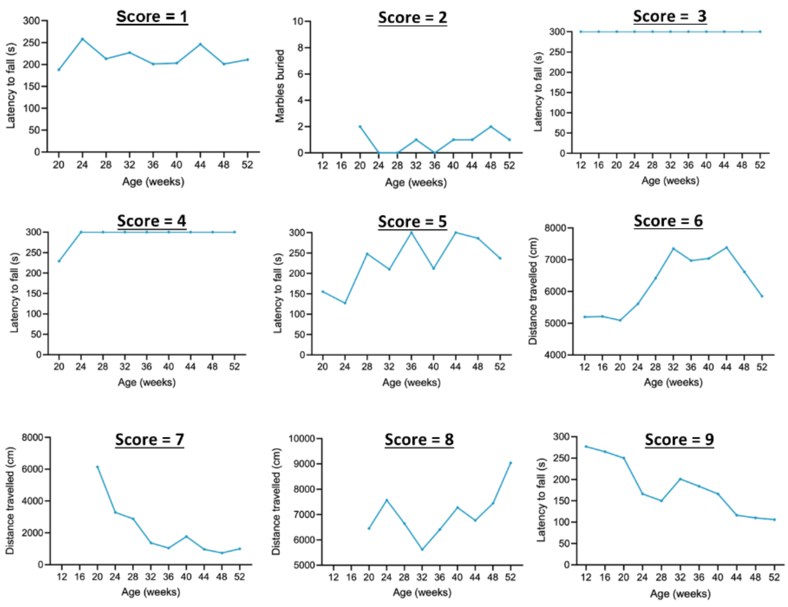

Disease Models & Mechanisms • Supplementary information

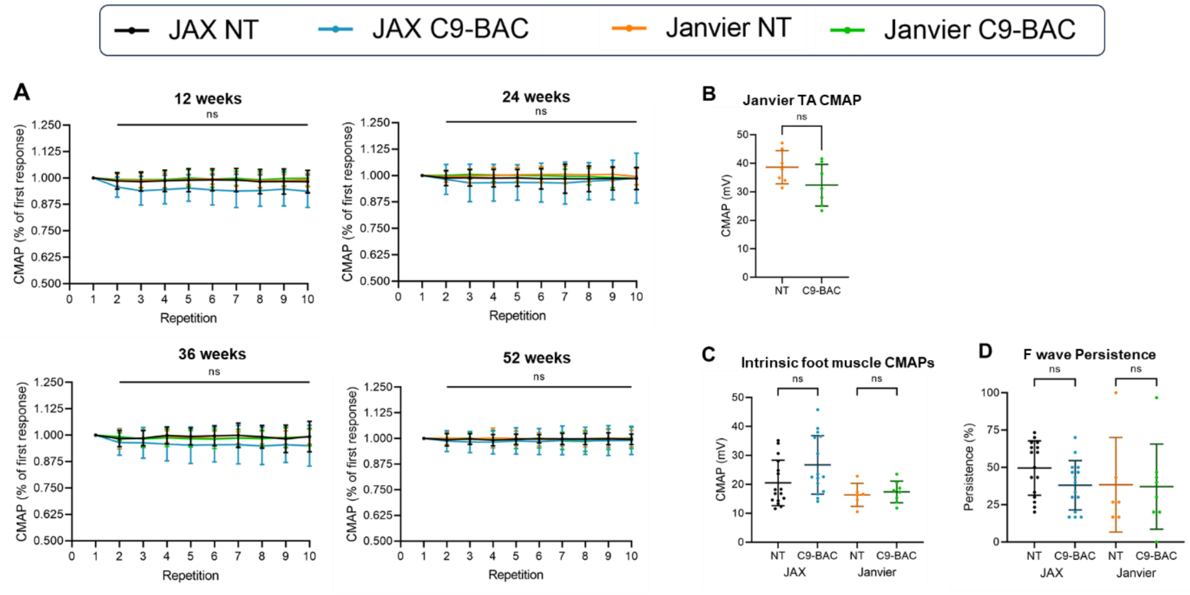

Fig. S7. No neuromuscular abnormalities in C9-BAC mice

A) Repetitive nerve stimulation tests (10 stimuli at 10 Hz) in the hindlimb muscles revealed no significant decrement in response (represented as percentage of the first response) in C9-BAC mice compared to NT littermates in both cohorts (JAX C9-BAC n = 14-32; NT n = 28-32; Janvier C9-BAC n = 28-32; NT n = 28-36; two-way ANOVA with repeated measures and Sidak's post-hoc test; ns, non-significant).

B) Compound muscle action potentials from the tibialis anterior muscle in C9-BAC and NT mice from the Janvier cohort at 52 weeks (n = 7 per group; unpaired t-test; ns, non-significant).

C) Compound muscle action potentials from the intrinsic foot muscles in a small group of C9-BAC and NT mice from both cohorts at 52 weeks (JAX C9-BAC n = 15; NT n = 16; Janvier C9-BAC n = 8; NT n = 6; unpaired t-test; ns, non-significant).

D) F wave persistence in intrinsic foot muscles of C9-BAC and NT mice from both cohorts at 52 weeks (JAX C9-BAC n = 15; NT n = 16; Janvier C9-BAC n = 8; NT n = 6; unpaired t-test; ns, non-significant). Values are mean ± SD.

Disease Models & Mechanisms • Supplementary information

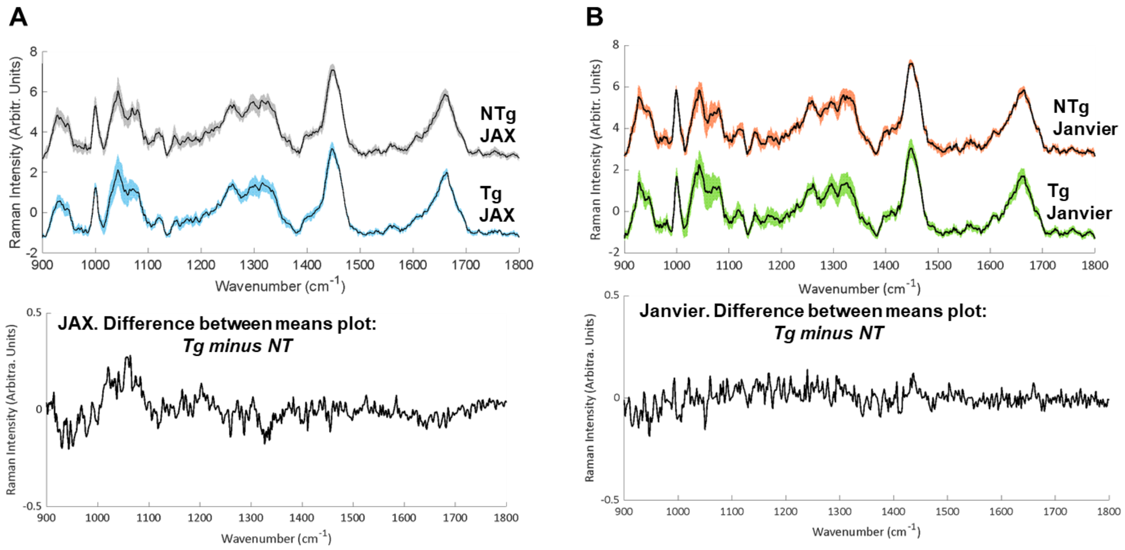

Fig. S8. No evidence of muscle pathology with Raman spectroscopy.

A) TOP: Mean (+/- standard deviation) Raman spectra for NT and C9-BAC JAX mice (JAX C9-BAC n= 12; NT = 12).

BOTTOM: Difference between the means plot. The low intensity peaks essentially represent noise.

B) TOP: Mean (+/- standard deviation) Raman spectra for NT and C9-BAC Janvier mice (Janvier C9-BAC n = 17; NT = 13).

BOTTOM: Difference between the means plot.

In addition, principal component fed linear discriminant analysis was attempted with up to 50 principal components (not shown). Utilising leave-one-out cross validation, no class (NT vs. C9-BAC) discrimination was seen with no accuracies above 50% (chance) observed.

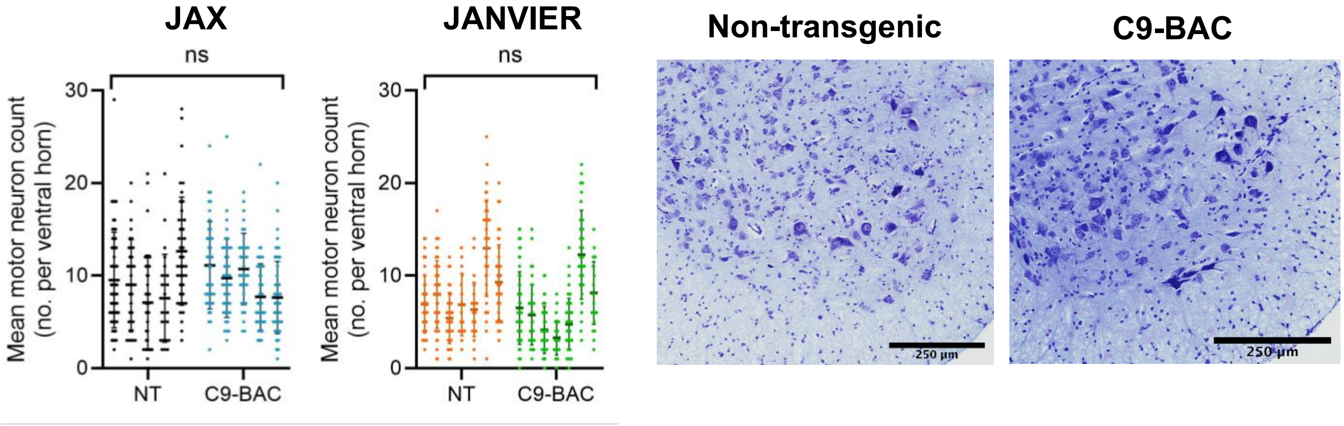

Fig. S9. No lower motor neuron loss in C9-BAC mice.

Left panel: motor neuron counts in ventral horns of the L4-5 region of the spinal cord at 52 weeks of age show no significant loss of motor neurons in C9-BAC mice in both cohorts (JAX n = 5 per group; Janvier n = 7 per group; mean ± SD; nested t-test). Right panel: representative examples of Nissl staining of motor neurons in the ventral horn of the lumbar spinal cord in NT and C9-BAC mice from the JAX cohort.

Disease Models & Mechanisms • Supplementary information

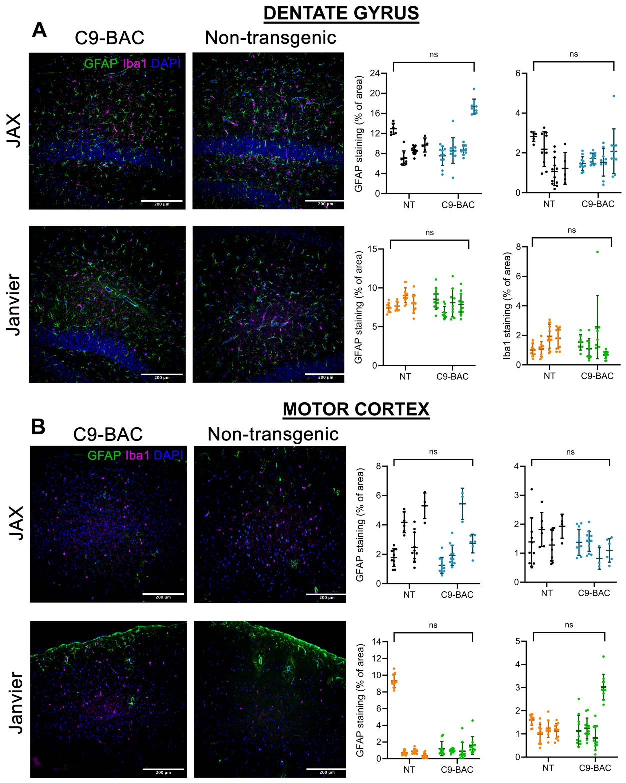

Fig. S10. Absence of pathological changes in the dentate gyrus and motor cortex of C9-BAC mice

A) GFAP and Iba1 staining in the dentate gyrus of JAX and Janvier mice at 52 weeks of age shows no evidence of astrogliosis or neuroinflammation (n = 4 per group).

B) GFAP and Iba1 staining of the motor cortex of JAX and Janvier mice at 52 weeks of age shows no evidence of astrogliosis or neuroinflammation (n = 4 per group).

Values are mean ± SD. Statistical comparisons performed by nested t-test.

Disease Models & Mechanisms • Supplementary information

Disease Models & Mechanisms • Supplementary information

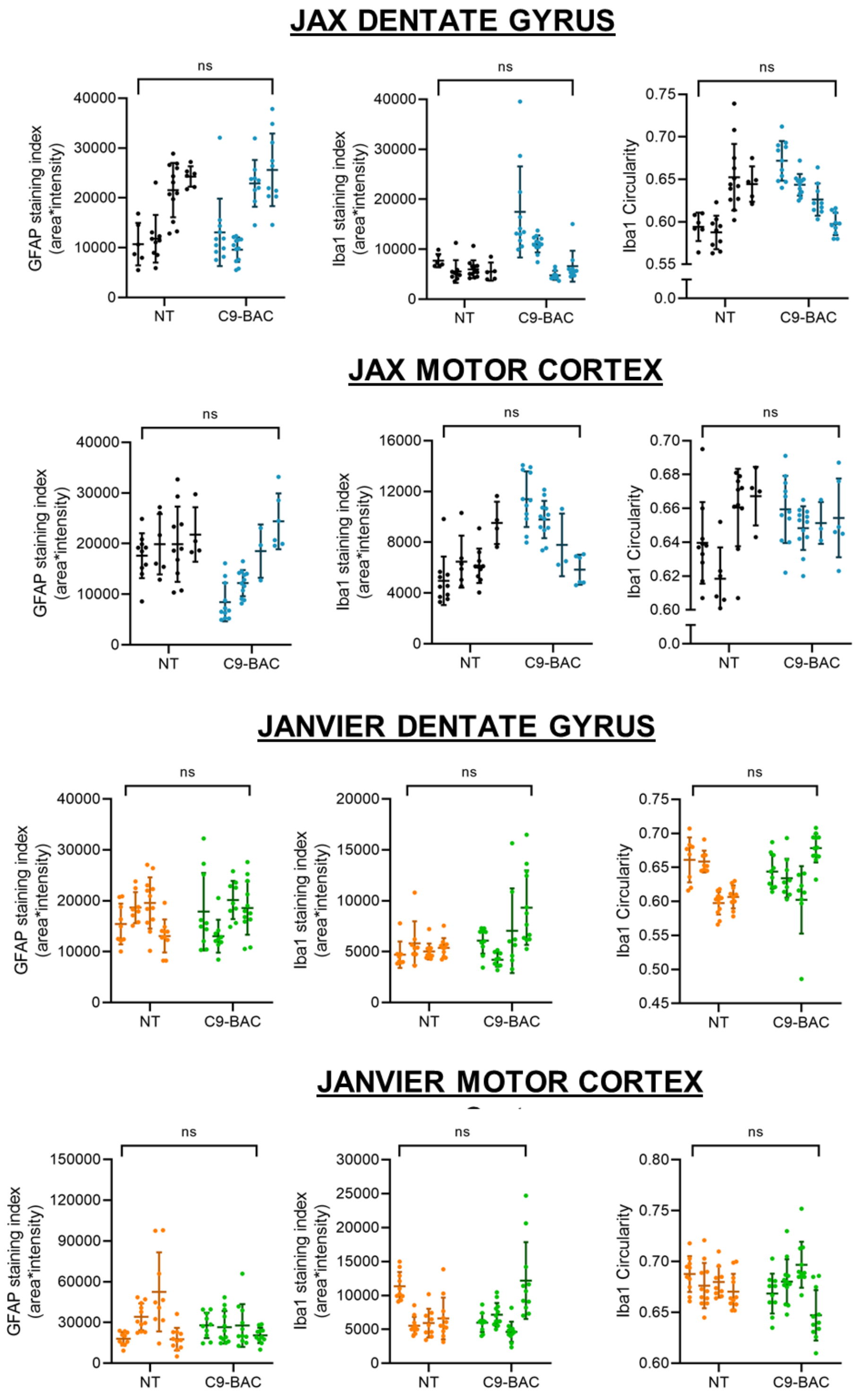

Fig. S11. Immunohistochemical Analysis of GFAP and Iba1 Staining in the Dentate Gyrus and Motor Cortex.

Further analysis of GFAP and Iba1 staining parameters in the dentate gyrus region of the hippocampus and the motor cortex. No significant differences were observed between C9-BAC mice and non-transgenic littermates in both cohorts (n = 4 per group). Values are mean ± SD. Statistical comparisons performed by nested t-test, ns = non-significant.

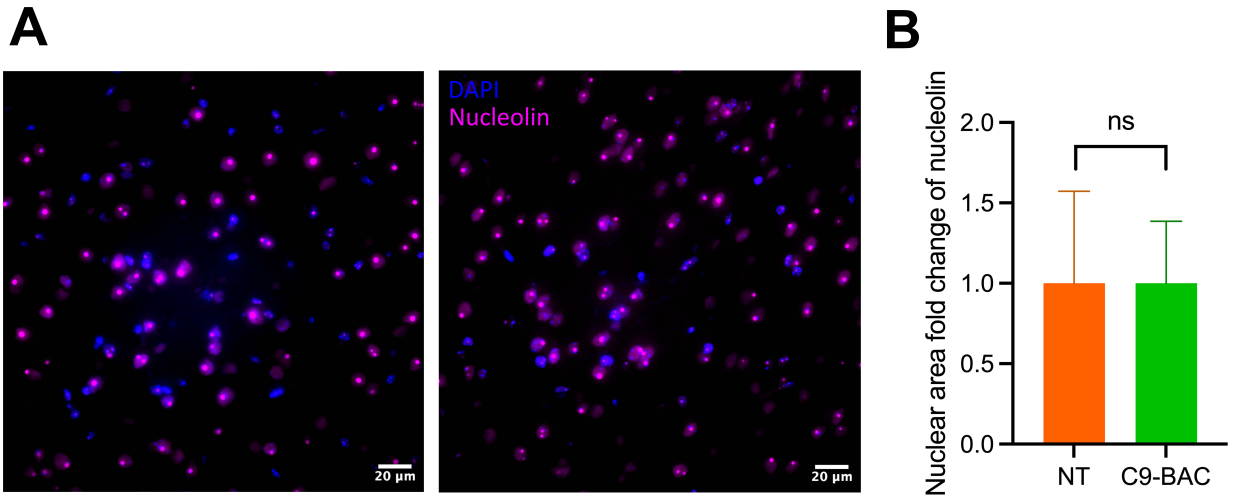

Fig. S12. No Evidence of Nucleolar Stress in the Motor Cortex of Janvier Mice

A) Nucleolin staining of the motor cortex shows no evidence of nucleolar stress in C9-BAC Janvier mice at 52 weeks of age.

B) Quantitation of nucleolin staining distribution showed no change in the ratio of the amount of staining in the nucleus vs. the nucleolus in the motor cortex (n=4 per group; nested t-test, NT vs. C9-BAC; ns, non-significant).

Disease Models & Mechanisms • Supplementary information

Table S1. Distress scoring. Animals with a cumulative score of 10–16 or a maximum score in three categories were considered to have reached humane endpoint and were euthanised. Weight loss was calculated by comparison with weight recorded at 12 weeks of age.

**Parameter**	**Description**	**Score**
**Weight loss**	0–10%	0
	10–19%	1
	>20%	3
**Appearance**	Normal	0
	Lack of grooming (dusty coat)	1
	Lack of grooming and hunched	2
**Behaviour**	Normal, bright, inquisitive	0
	Stereotypic behaviour (circling)	1
	Mostly inactive, responds if provoked	2
	Inactive, does not respond if provoked	4
**Clasping**	Both hind limbs splay normally	0
	One hind limb splays normally	1
	Both hind limbs close to abdomen	2
	Both hind limbs tightly clasped	4
**Neurologic**	Normal	0
	Immobile and staring	1
	Seizure (<5 seconds) with recovery **or** hindlimb paralysis	2
	Seizure (>10 seconds) **or** any seizure plus hind limb paralysis	4

Disease Models & Mechanisms • Supplementary information

Table S2. Subgroup analysis: contingency table counts of C9-BAC and NT mice.
Each number represents the number of times a score was given for each test. Fisher's exact test was performed between C9-BAC and NT groups in each cohort. No evidence of transgene-related subsets within the data were found.

	Test	C9-BAC		Non-transgenic		p-value
		Potential ALS-FTD phenotype	Non-ALS/FTD phenotype	Potential ALS-FTD phenotype	Non-ALS/FTD phenotype	
JAX	**Rotarod**	14	18	11	23	0.45
	**Open field**	10	22	9	25	0.79
	**Marble Burying**	21	11	18	16	0.33
	**Balance Beam**	17	15	18	16	>0.99
	**Limb Hang**	14	18	13	21	0.80
	**Burrowing**	16	16	14	20	0.62
	**Nesting**	12	20	15	19	0.62
	**CMAP**	11	21	12	22	>0.99
Janvier	**Rotarod**	8	24	4	24	0.35
	**Open field**	6	26	9	19	0.25
	**Marble Burying**	13	19	13	15	0.79
	**Balance Beam**	14	18	12	15	>0.99
	**Limb Hang**	13	19	16	12	0.30
	**Burrowing**	15	17	15	13	0.80
	**Nesting**	14	18	13	15	>0.99
	**CMAP**	17	15	15	13	>0.99

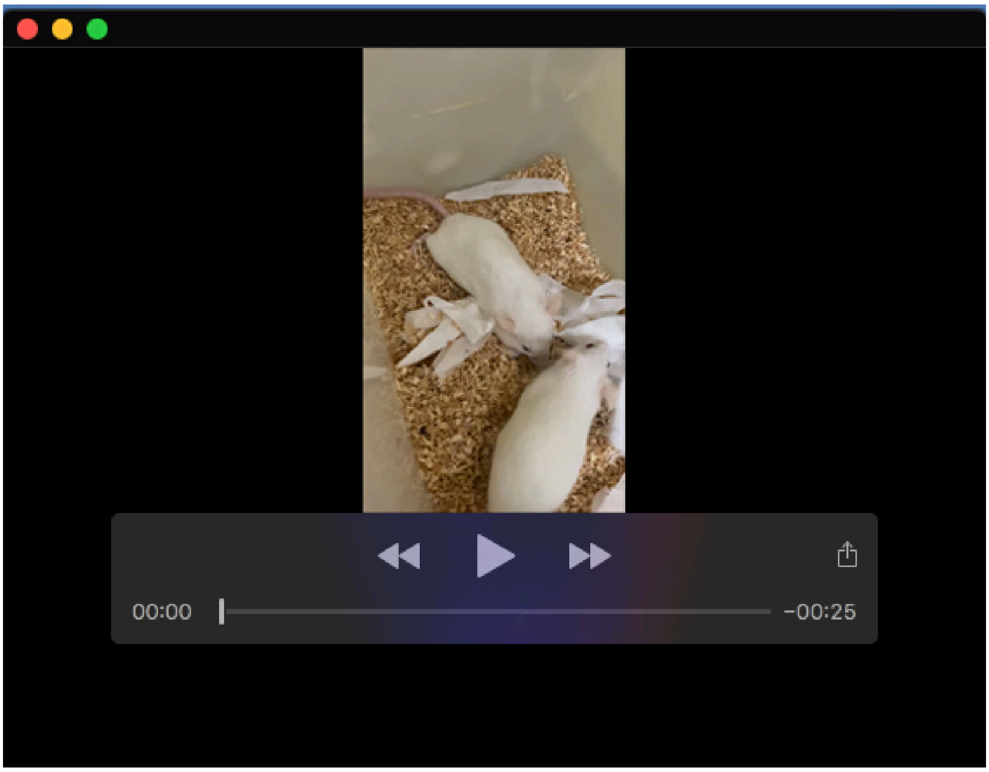

Movie 1. Example of a NT mouse post-seizure displaying laboured breathing and lack of reactivity when provoked.

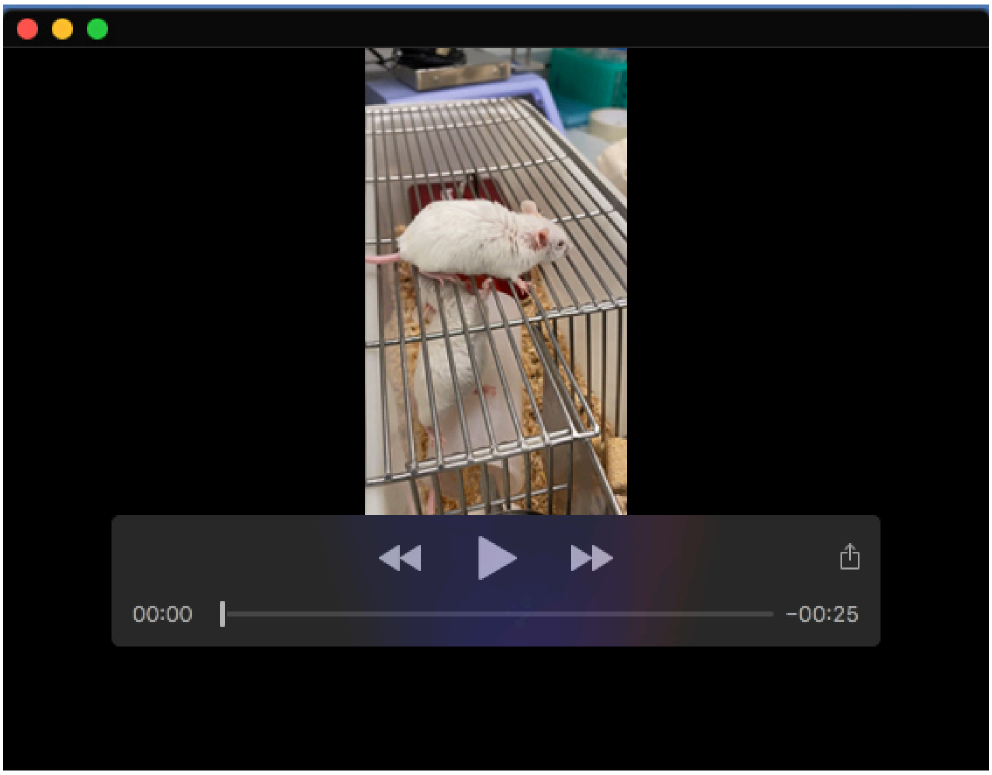

Movie 2. Example of a C9-BAC mouse post-seizure displaying laboured breathing, reduced activity, orbital tightening, change in ear shape/position, piloerection. When picked up hindlimb clasping is evident.

Supplementary Materials and Methods

Open field test

Mice were placed in a grey acrylic chamber (45 cm x 45 cm x 45 cm) under white light. Their behaviour was video-recorded and analysed using Smart Video Tracking Software V3.0 (Panlab, Harvard Apparatus) for 10 minutes. Distance travelled and time spent in the centre zone (25 cm x 25 cm) and periphery zone of the chamber were examined to measure locomotion and levels of anxiety, respectively.

Burrowing test

Burrowing tubes (7 cm x 7 cm x 20 cm) were made from plastic piping and sealed at one end. The open end of the burrowing tube was elevated by approximately 1 cm using machine screws (5 cm) inserted 1 cm from the opening. Individual mice were placed into a cage (42.5 cm x 26.6 cm x 18.5 cm) lined with sawdust (~1 cm) and access to food and water ad libitum. A burrowing tube was filled with 200 g of food pellets (2018 Teklad global rodent diet, Envigo) and placed into the cage. After two hours, a baseline measurement was taken. The burrow was emptied and the food pellets weighed before being replaced into the burrow and returned to the cage. This weighing procedure was completed even if no pellets had been burrowed out of the tube to provide a consistent environment. The weight burrowed was calculated by subtracting from 200 g. The final reading was taken the following morning.

Nesting

Individual mice were placed in a cage (42.5 cm x 26.6 cm x 18.5 cm) filled with a thin layer (~1 cm) of sawdust and access to food and water ad libitum. A 'nestlet' (2 inches x 2 inches, Ancare) was weighed and placed in the centre of each cage. The following morning, the nest was scored using criteria detailed previously (Deacon, 2012) and the amount of untorn nestlet weighed.

Balance beam

Mice were placed on a cage ledge and observed walking along it. Each mouse was scored using criteria detailed previously (Guyenet et al., 2010). Each mouse underwent three trials and the median score from the trials was taken for analysis.

Catwalk

The catwalk gait analysis system 7.1 (Noldus Information Technology B.V., Netherlands) was used to capture gait parameters. Mice were placed onto the catwalk and allowed to walk freely in total darkness. 6 straight, continuous runs were recorded, and the 3 best runs were chosen for analysis.

The Catwalk Software 7.1 was used to manually label each paw print of a run to analyse the gait of each mouse. Data were collated using Microsoft Excel and informative parameters chosen for analysis.

Social recognition test

Mice were placed into a grey acrylic chamber (45 cm x 45 cm x 45 cm) under white light with an empty mesh cage in the centre immediately prior to testing and allowed to habituate to the environment for 10 minutes. The mouse was removed from the chamber and a juvenile NT female mouse was placed into the mesh cage in the centre. The adult mouse was returned to the chamber for the initial interaction trial of 2 minutes. The trial was repeated with the same juvenile female mouse 3 days later (recall session), followed by a trial with an unfamiliar juvenile NT female mouse (novel session). Each trial was video-recorded and analysed using Smart Video Tracking Software V3.0 (Panlab, Harvard Apparatus). Total time spent investigating the juvenile mouse was calculated and used for analysis. Investigation was defined as any contact exceeding 1 second. Recognition index was calculated as time spent investigating in the recall session relative to time spent investigating in the initial session.

Subset analysis

To identify possible latent subsets within the data, a method was devised to assess the performance of each mouse in each behavioural test. A scoring system based on cohort quartiles was created to describe nine different data patterns (detailed in supplemental figure). In brief, scores which could be compatible with a neurodegenerative phenotype were deemed to be those in which performance in a given test was either in the bottom quartile throughout testing or declined sufficiently to drop into the quartile below. Thus, such scores represent consistent dysfunction, or a deterioration in function without improvement and were considered potential ALS/FTD phenotypes. To execute this, the median and interquartile ranges were calculated for each behavioural test and the performance of each mouse in each test was checked at each timepoint against these values and a score assigned. The number of times each score was assigned per test was then tallied for C9-BAC and NT groups. The number of times each potential ALS/FTD phenotype score and non-ALS/FTD score was assigned per test was then tallied for C9-BAC and NT groups and a Fisher's exact test performed.

Nissl staining

For nissl staining slides were deparaffinised in two changes of xylene and rehydrated through graded ethanol, submerged in 0.25% cresyl violet for 2 minutes, and differentiated in 0.25% acetic acid in alcohol for 10 seconds. Slides were briefly immersed in 100% ethanol, cleared in xylene, and coverslipped using DPX mounting media. Slides were visualised using a digital slide scanner

(NanoZoomer XR, Hamamatsu). Motor neuron counting was performed blinded to genotype using Qupath open-source software (Bankhead et al., 2017) on 4 slides per mouse, with 8 sections per slide. Sections on each slide were separated by 50 μm to prevent double counting. Motor neurons were identified only by a clearly visible, nissl-stained nucleolus and a soma size larger than 25 μm. Any sections with damage or poor staining were excluded. Number of motor neurons per ventral horn per section were analysed on Graphpad Prism 9 using a nested t-test.

Nucleolin staining

Sections were immunostained by incubating in primary antibody for nucleolin (1:1000, Cat #ab22758, Abcam) overnight at 4°C and then incubating in appropriate fluorescent secondary antibody (1:1000, A27039) for 90 minutes. Slides were coverslipped using Hardset Vectashield with 4',6-diamidino-2-phenylindole (DAPI) mounting medium (Vectorlabs) and imaged using an IN-Cell Analyzer 2000 (GE Healthcare). Images were taken at 60x magnification. 5 random images from the motor cortex were obtained from 4 non-transgenic age-matched controls and 4 C9-BAC mice at 52 weeks of age from the Janvier colony. Nucleolin displacement was calculated using ImageJ software as follows (Total nucleolin – nucleolin in nucleolus/nuclear area – nucleolar area = nucleolin dispersed in the nucleus)(O'Rourke et al., 2015) and analysed using a nested t-test.

Raman spectroscopy

The in vivo fiber optic methodology was undertaken as previous (Plesia et al., 2021). Briefly, mice were anesthetized with 2% isoflurane, hindlimbs were shaved and the fiber optic Raman needle probe inserted into the medial and lateral heads of gastrocnemius bilaterally (thus obtaining four spectra per mouse). The incident light was provided by an 830-nm laser (power output 60 mW at the probe tip) and the probe optically paired to the spectrometer. The Raman signal was recorded by averaging 10x 4-second epochs (total recording time of 40 seconds for each placement of the probe).

Analysis was done in MATLAB 2019a (The MathWorks) using custom scripts. Raman spectra were interpolated and windowed in the biological fingerprint region between 900 cm^{-1} and 1800 cm^{-1} to avoid silica background in the optical fibers (at <900 cm^{-1}) and uninformative noise >1800 cm^{-1}. Spectra were background using the rubberband algorithm (Paraskevaidi et al., 2017), smoothed (second-order Savitzky-Golay filter, 5-datapoint window width) and then vector normalised. Spectra are shown arbitrarily offset on the y-axis for easy of presentation. Difference between the mean plots were obtained by calculating mean spectra for the C9-BAC and non-transgenic groups and then subtracting one mean from the other. For principal component fed-linear discriminant analysis data were mean centred and principal component analysis performed. Up to 50 principal components were then fed into the linear discriminant analysis and the ensuing model cross validated using a leave-one-mouse-out approach.

Disease Models & Mechanisms • Supplementary information

Supplementary References

Bankhead, P., Loughrey, M. B., Fernández, J. A., Dombrowski, Y., McArt, D. G., Dunne, P. D., McQuaid, S., Gray, R. T., Murray, L. J., Coleman, H. G., James, J. A., Salto-Tellez, M. & Hamilton, P. W. (2017). QuPath: Open source software for digital pathology image analysis. *Scientific Reports.* **7** (1), 16878. doi: 10.1038/s41598-017-17204-5.

Deacon, R. (2012). Assessing burrowing, nest construction, and hoarding in mice. *J Vis Exp.* (59), e2607. doi: 10.3791/2607.

Guyenet, S. J., Furrer, S. A., Damian, V. M., Baughan, T. D., La Spada, A. R. & Garden, G. A. (2010). A simple composite phenotype scoring system for evaluating mouse models of cerebellar ataxia. *J Vis Exp.* (39). doi: 10.3791/1787.

O'Rourke, J. G., Bogdanik, L., Muhammad, A., Gendron, T. F., Kim, K. J., Austin, A., Cady, J., Liu, E. Y., Zarrow, J., Grant, S., Ho, R., Bell, S., Carmona, S., Simpkinson, M., Lall, D., Wu, K., Daughrity, L., Dickson, D. W., Harms, M. B., Petrucelli, L., Lee, E. B., Lutz, C. M. & Baloh, R. H. (2015). C9orf72 BAC Transgenic Mice Display Typical Pathologic Features of ALS/FTD. *Neuron.* **88** (5), 892-901. doi: 10.1016/j.neuron.2015.10.027.

Paraskevaidi, M., Morais, C. L. M., Lima, K. M. G., Snowden, J. S., Saxon, J. A., Richardson, A. M. T., Jones, M., Mann, D. M. A., Allsop, D., Martin-Hirsch, P. L. & Martin, F. L. (2017). Differential diagnosis of Alzheimer's disease using spectrochemical analysis of blood. *Proc Natl Acad Sci U S A.* **114** (38), E7929-E7938. doi: 10.1073/pnas.1701517114.

Plesia, M., Stevens, O. A., Lloyd, G. R., Kendall, C. A., Coldicott, I., Kennerley, A. J., Miller, G., Shaw, P. J., Mead, R. J., Day, J. C. C. & Alix, J. J. P. (2021). In Vivo Fiber Optic Raman Spectroscopy of Muscle in Preclinical Models of Amyotrophic Lateral Sclerosis and Duchenne Muscular Dystrophy. *ACS Chem Neurosci.* **12** (10), 1768-1776. doi: 10.1021/acschemneuro.0c00794.
